# Supplementary material for: Design, Synthesis, Docking Study, and Antiproliferative Evaluation of Novel Schiff Base–Benzimidazole Hybrids with VEGFR-2 Inhibitory Activity
Source: Molecules. 2023 Jan 4;28(2):481. doi: 10.3390/molecules28020481 (PMC9862622; doi:10.3390/molecules28020481)
Supplement: Supplementary file 1 [file molecules-28-00481-s001.zip › molecules-2104860-supplementary.pdf]

Supporting Information

Article

Design, Synthesis, Docking Study, and Antiproliferative Evaluation of Novel Schiff Base-Benzimidazole Hybrids with VEGFR-2 Inhibitory Activity

Hany M. Abd El-Lateef [1,2,*], Mohammed A. I. Elbastawesy [3,*], Tamer Mohamed Abdelghani Ibrahim [4,5], Mai M. Khalaf [1,2], Mohamed Gouda [1], Mariam G. F. Wahba [6], Islam Zaki [7] and Martha M. Morcoss [8]

[1] Department of Chemistry, College of Science, King Faisal University, Al-Ahsa 31982, Saudi Arabia; mmkali@kfu.edu.sa (M.M.K.); mgoudaam@kfu.edu.sa (M.G.)
[2] Department of Chemistry, Faculty of Science, Sohag University, Sohag 82524, Egypt
[3] Department of Pharmaceutical Organic Chemistry, Faculty of Pharmacy, Al-Azhar University, Assiut 71524, Egypt
[4] Community Engagement Development Administration, Vice-Presidency for Studies, Development and Community Service, King Faisal University, Al-Ahsa 31982, Saudi Arabia; tabdelghani@kfu.edu.sa
[5] Faculty of Social Work, Helwan University, Helwan 11795, Egypt, tamer_abdelghani@social.helwan.edu.eg
[6] Department of Pharmacology and Toxicology, Faculty of pharmacy, Nahda University, Beni-Suef 62513, Egypt; mariam.gamal@nub.edu.eg
[7] Pharmaceutical Organic Chemistry Department, Faculty of Pharmacy, Port Said University, Port Said 42526, Egypt; eslam.zaki@pharm.psu.edu.eg
[8] Department of Pharmaceutical Chemistry, Faculty of Pharmacy, Nahda University, Beni-Suef 62513, Egypt; martha.moheb@nub.edu.eg
* Correspondence: hmahmed@kfu.edu.sa (H.M.A.E.-L.); mohamedali.pharm.ast@azhar.edu.eg (M.A.I.E.); Tel.: +20-100-429-2549 (M.A.I.E.)

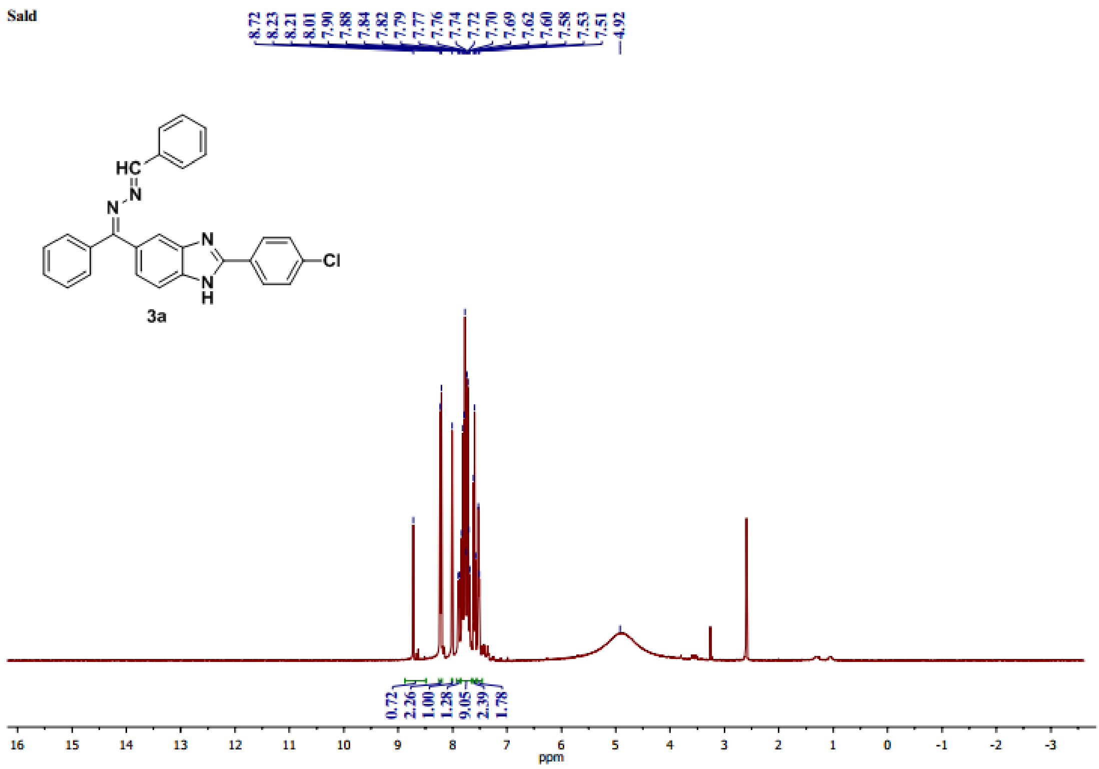

Figure S1: ^{1}H-NMR spectrum of compound **3a**

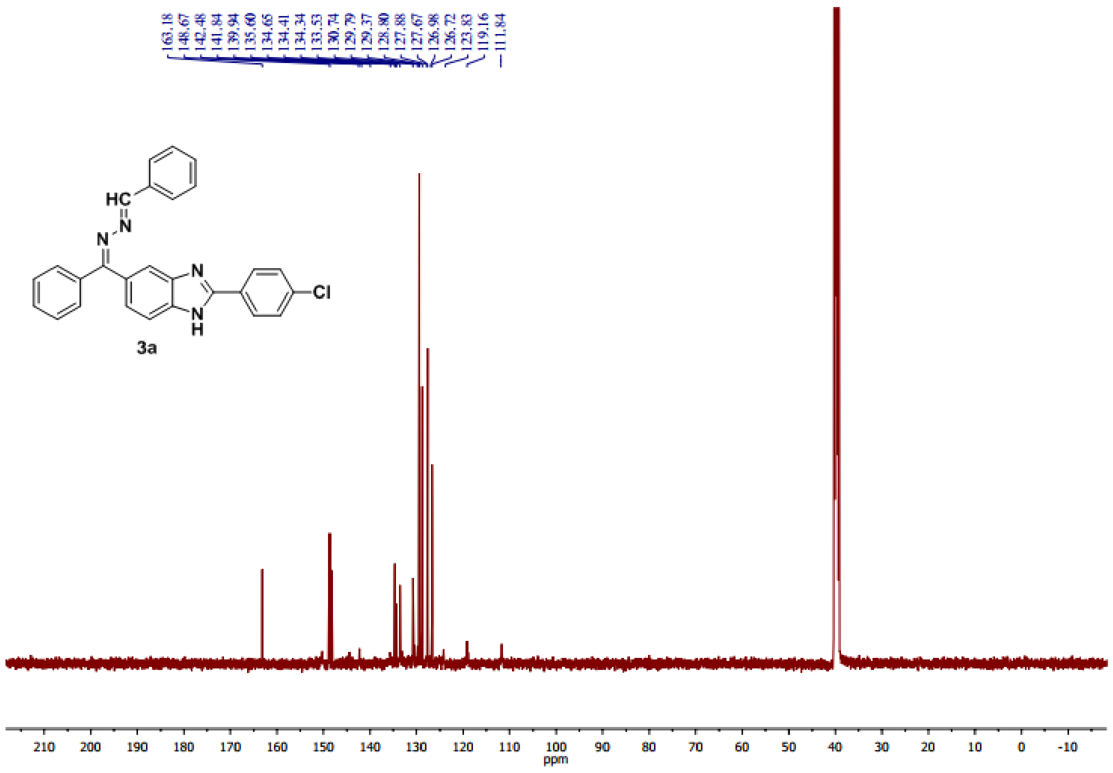

Figure S2: ^{13}C-NMR spectrum of compound **3a**

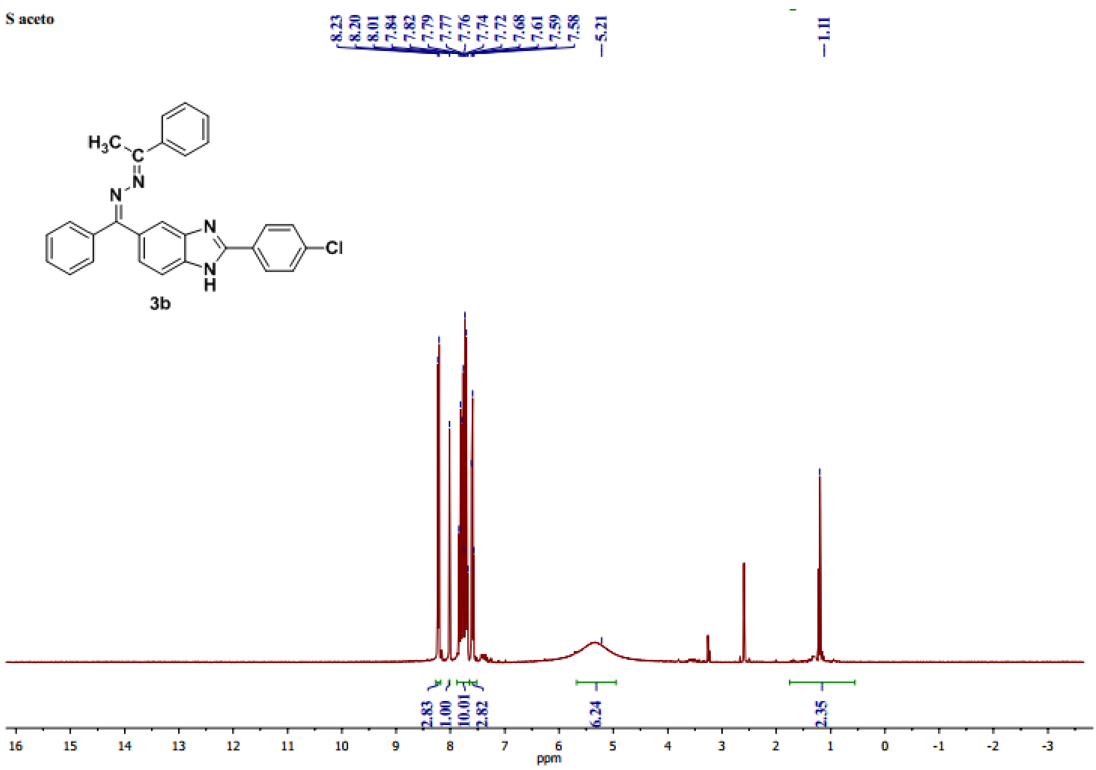

Figure S3: ^{1}H-NMR spectrum of compound **3b**

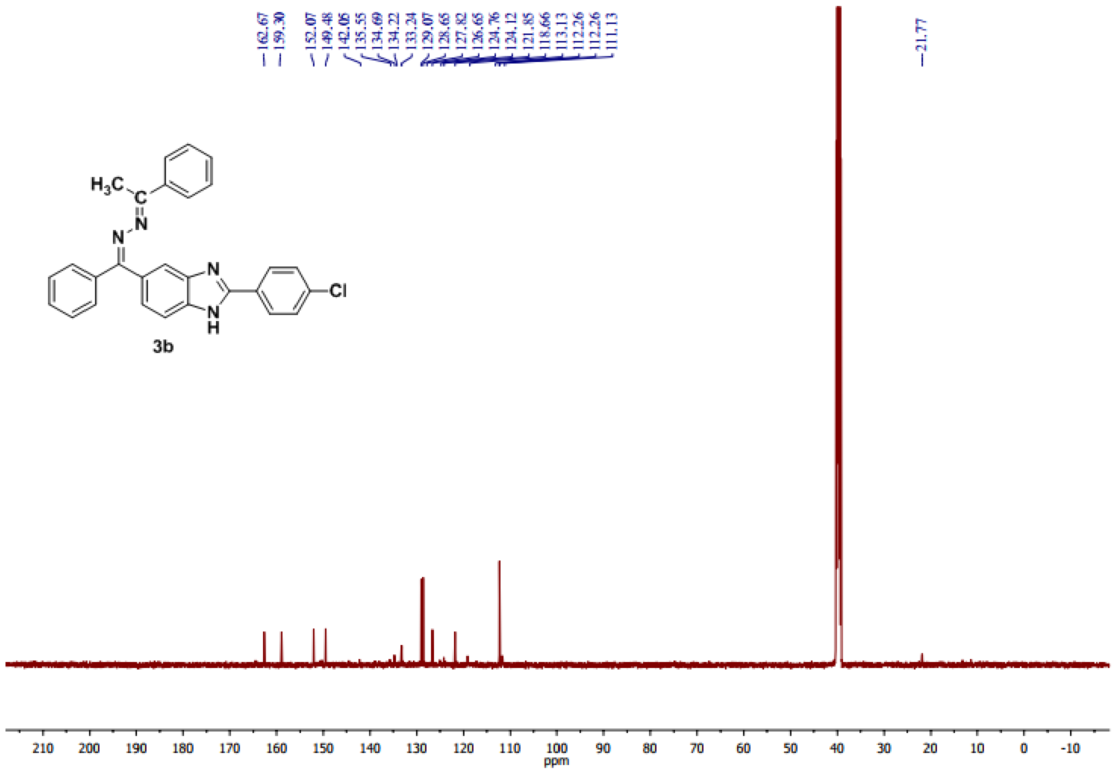

Figure S4: ^{13}C-NMR spectrum of compound **3b**

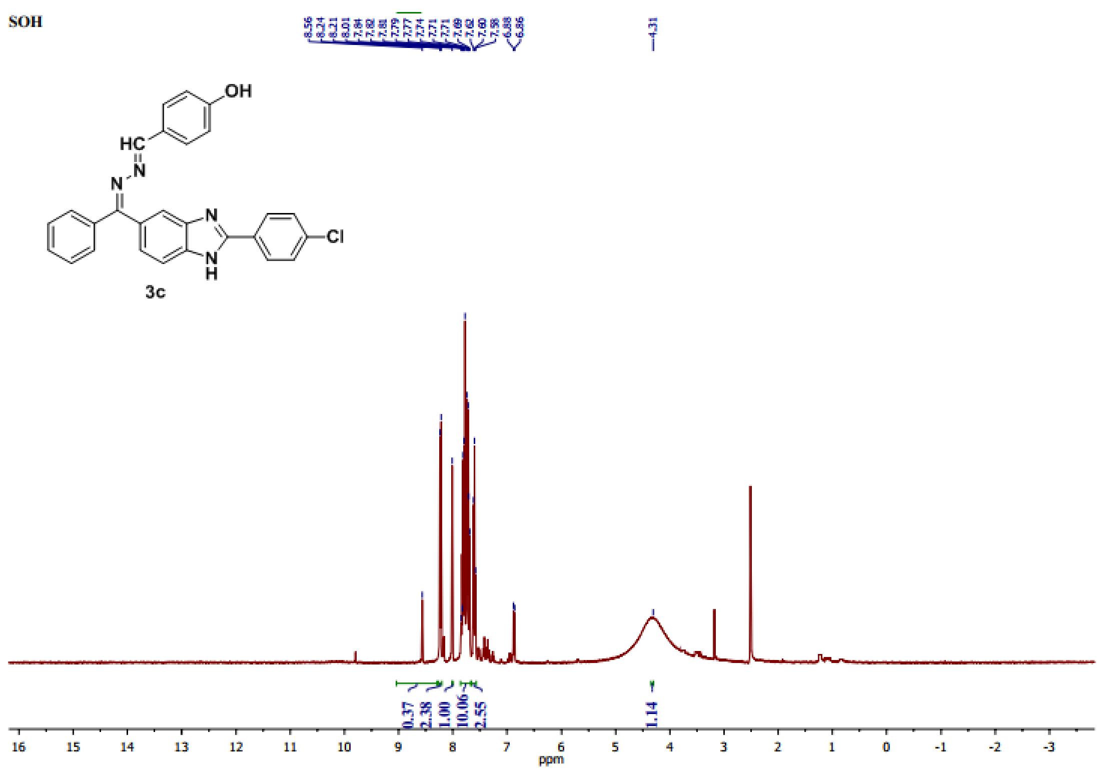

Figure S5: ^{1}H-NMR spectrum of compound **3c**

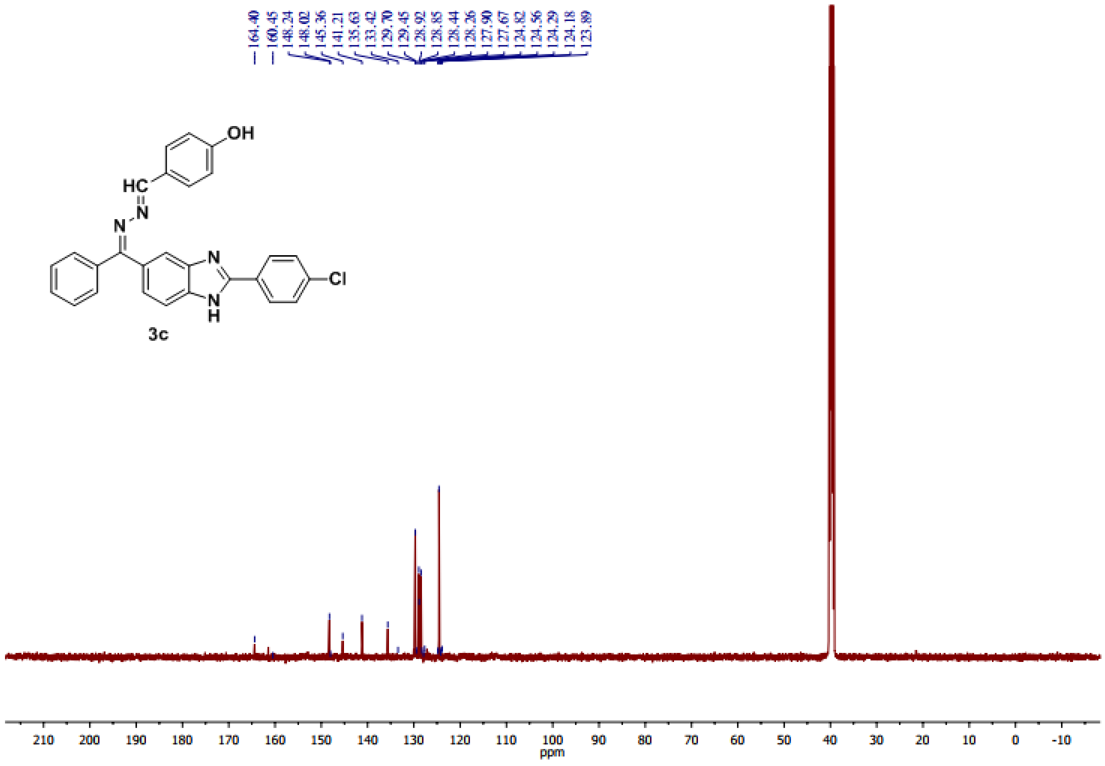

Figure S6: ^{13}C-NMR spectrum of compound **3c**

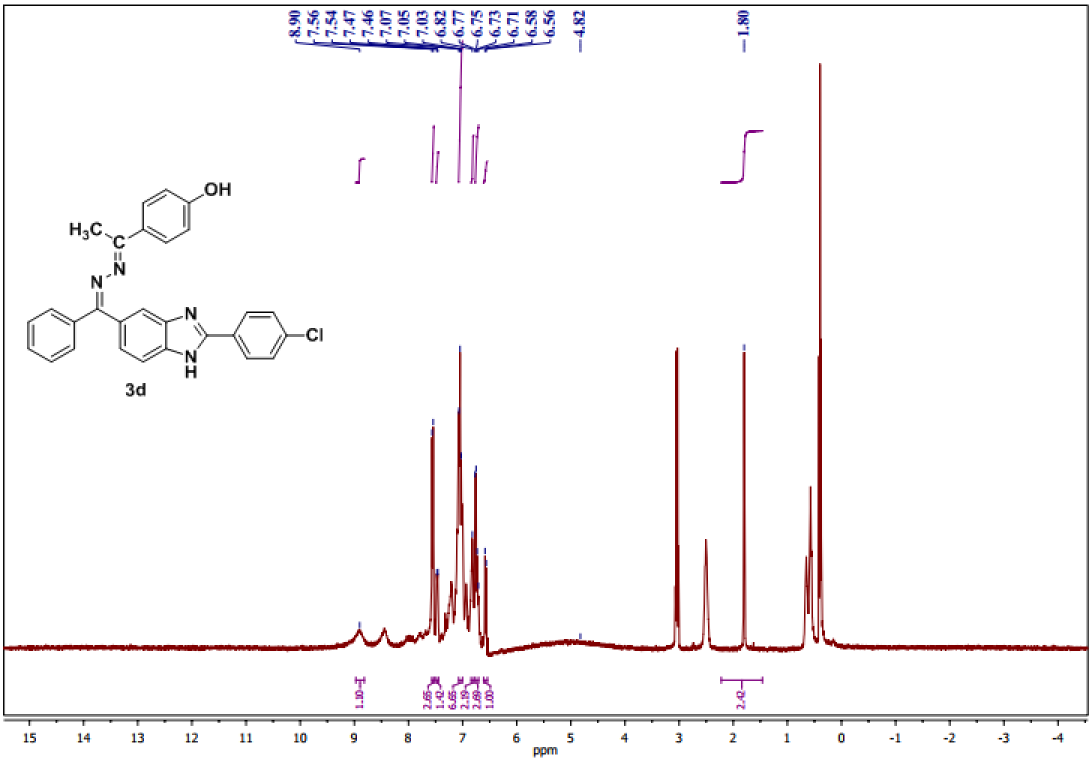

Figure S7: ^{1}H-NMR spectrum of compound **3d**

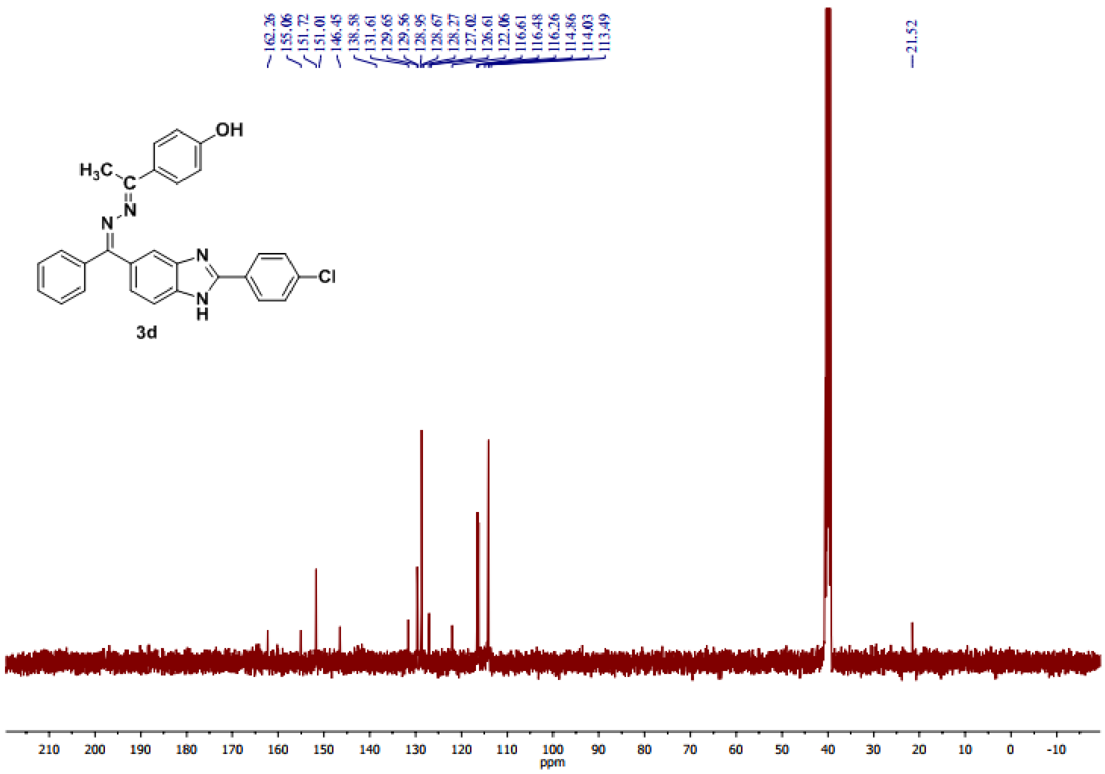

Figure S8: ^{13}C-NMR spectrum of compound **3d**

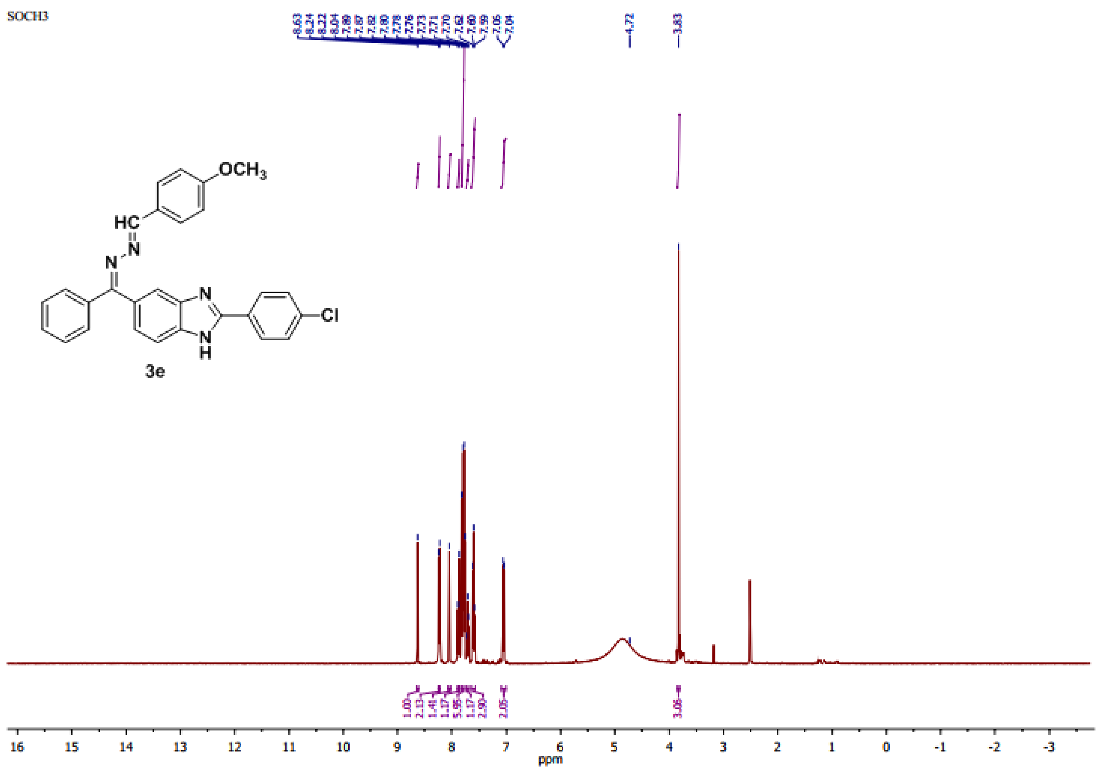

Figure S9: ^{1}H-NMR spectrum of compound **3e**

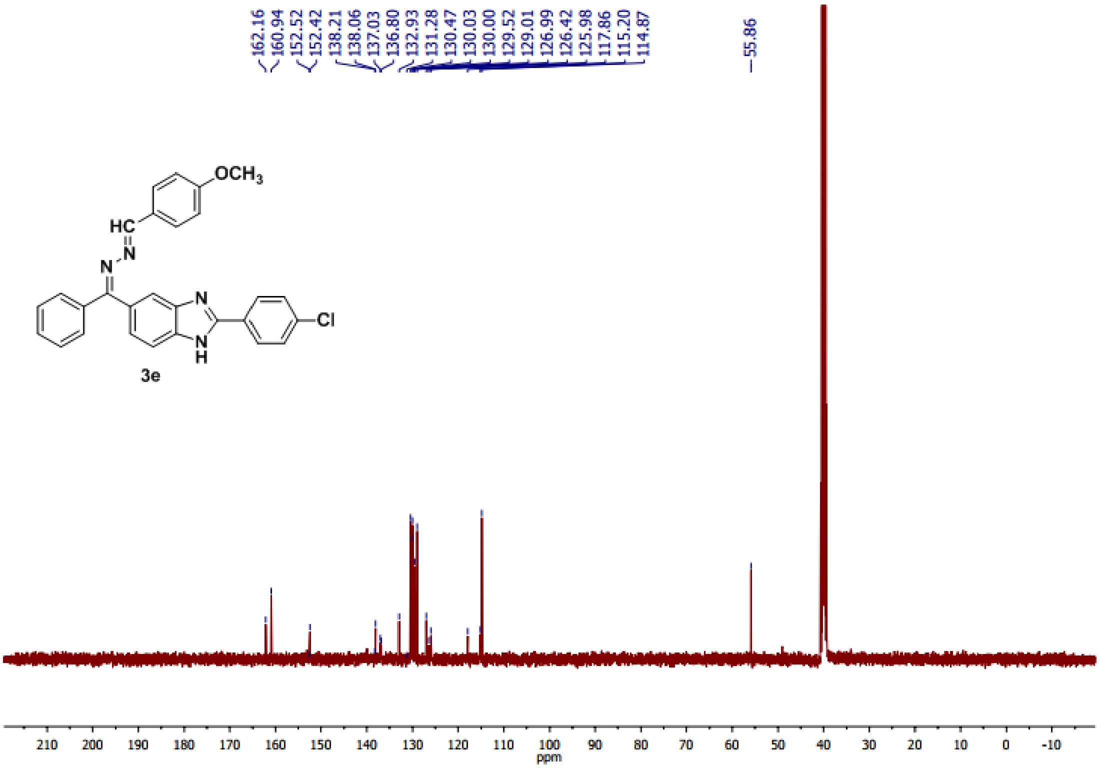

Figure S10: ^{13}C-NMR spectrum of compound **3e**

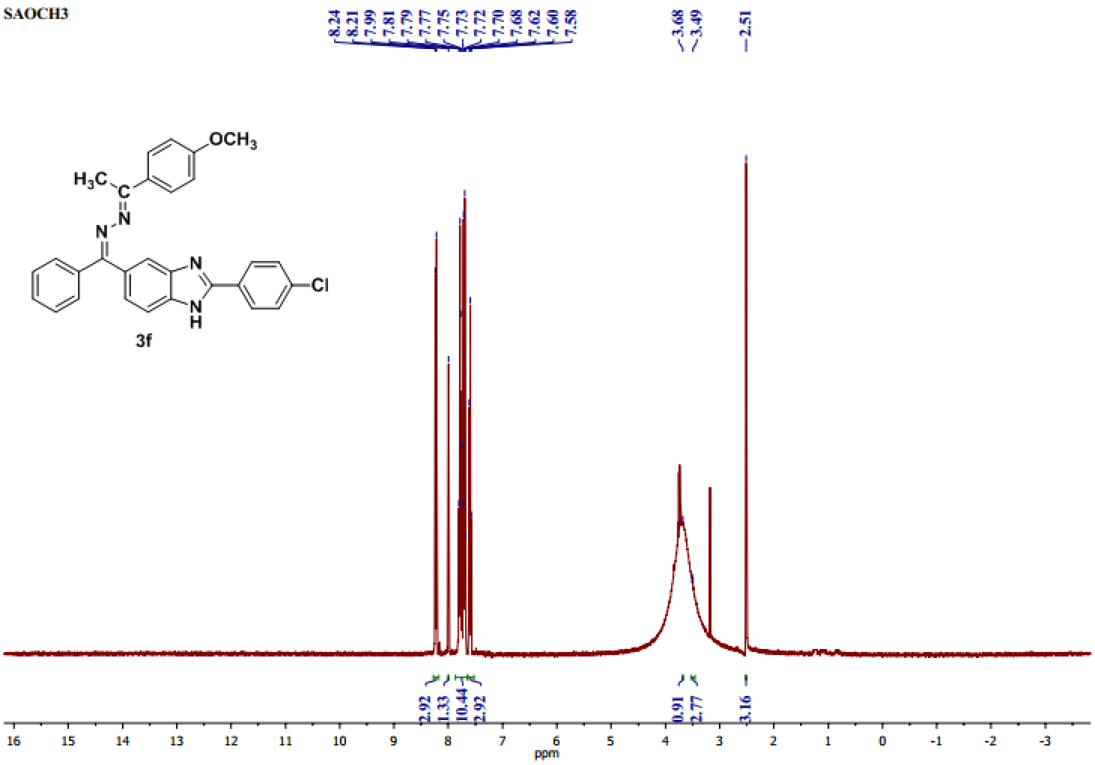

Figure S11: ^{1}H-NMR spectrum of compound **3f**

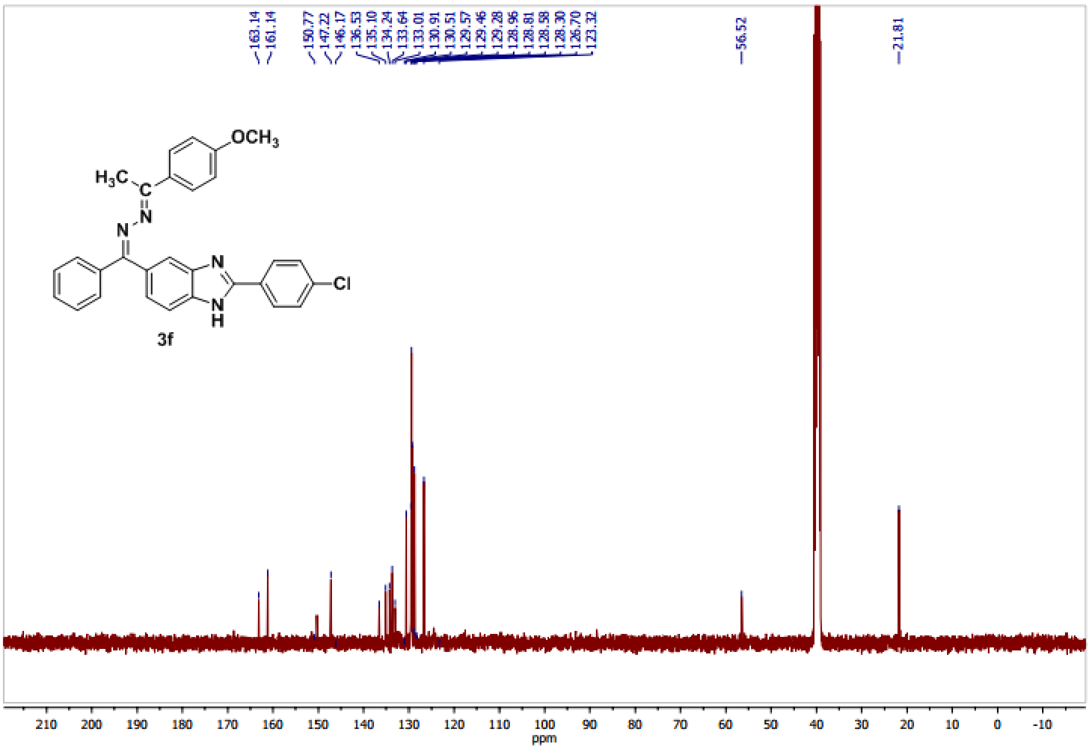

Figure S12: ^{13}C-NMR spectrum of compound **3f**

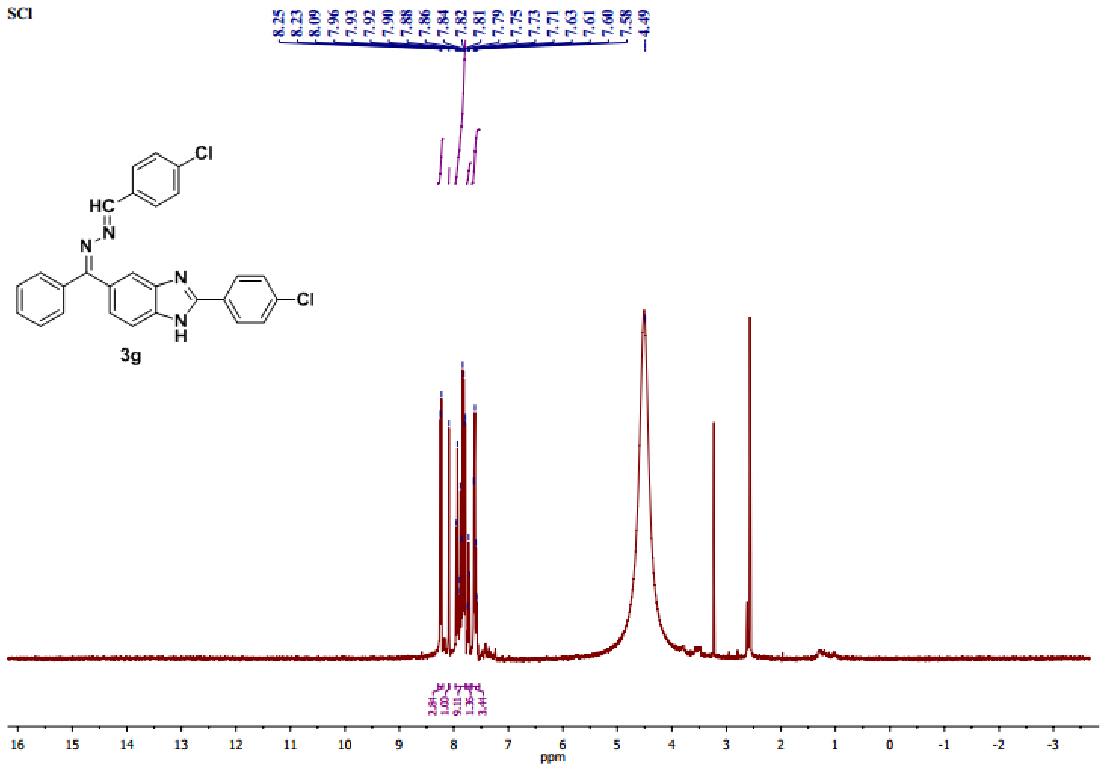

Figure S13: ^{1}H-NMR spectrum of compound **3g**

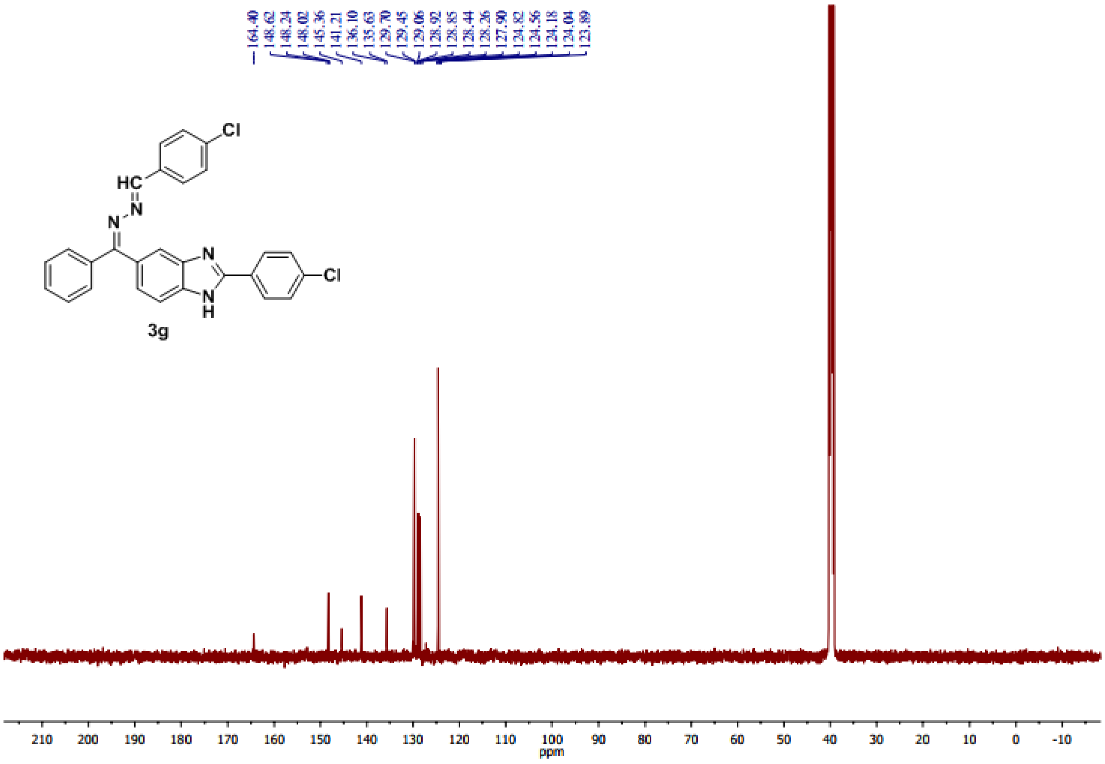

Figure S14: ^{13}C-NMR spectrum of compound **3g**

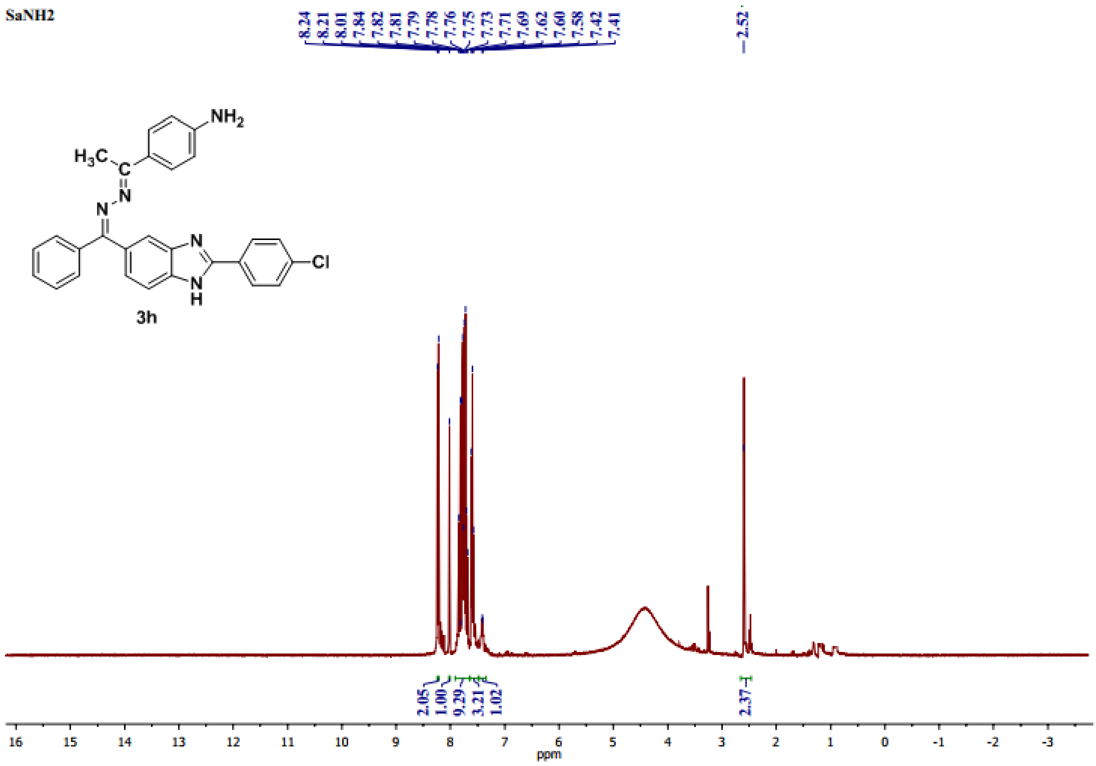

Figure S15: ^{1}H-NMR spectrum of compound **3h**

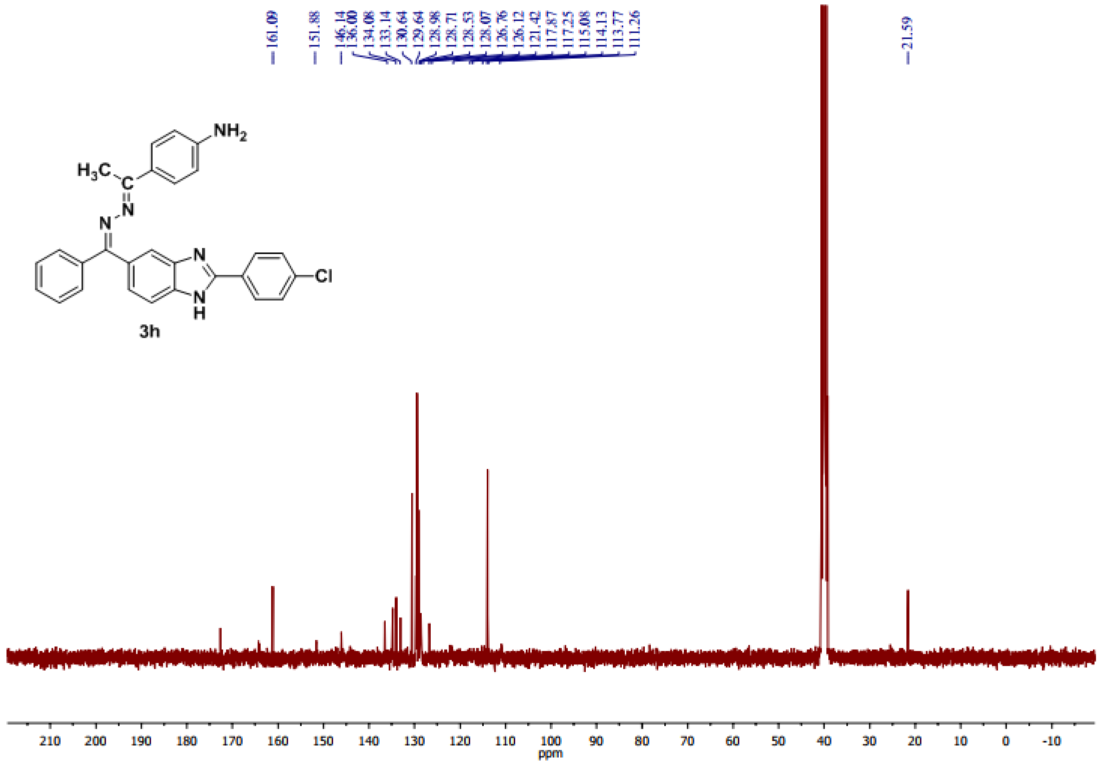

Figure S16: ^{13}C-NMR spectrum of compound **3h**

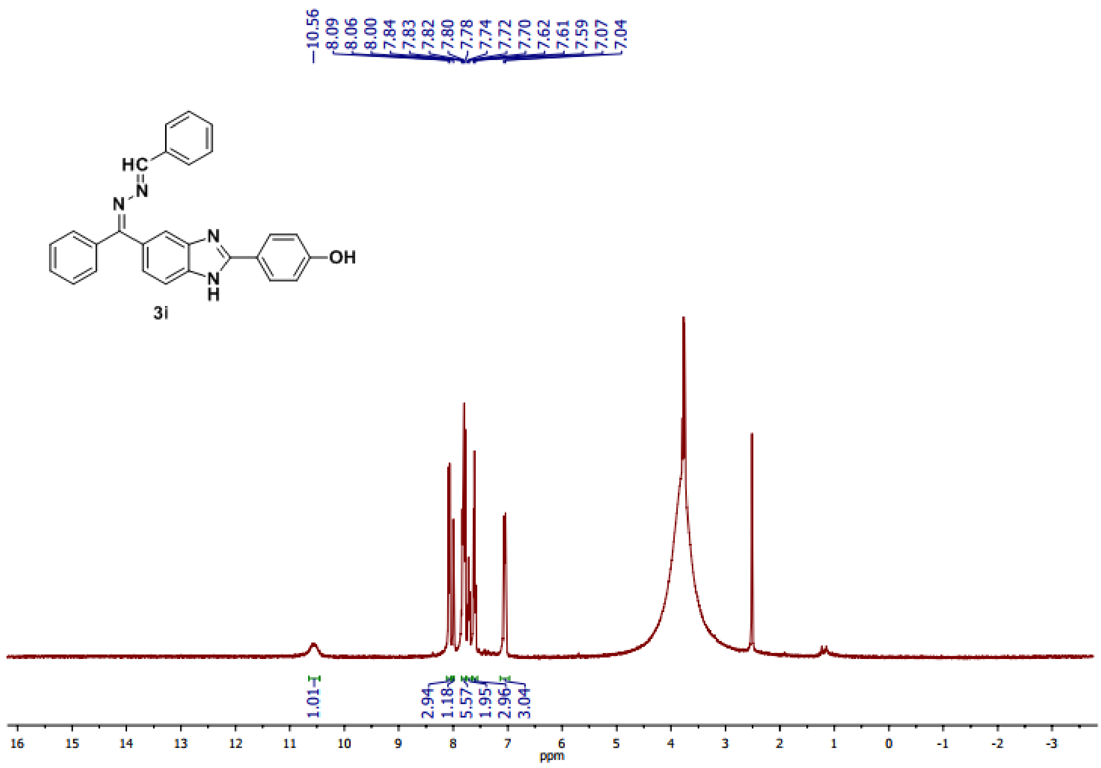

Figure S17: ^{1}H-NMR spectrum of compound **3i**

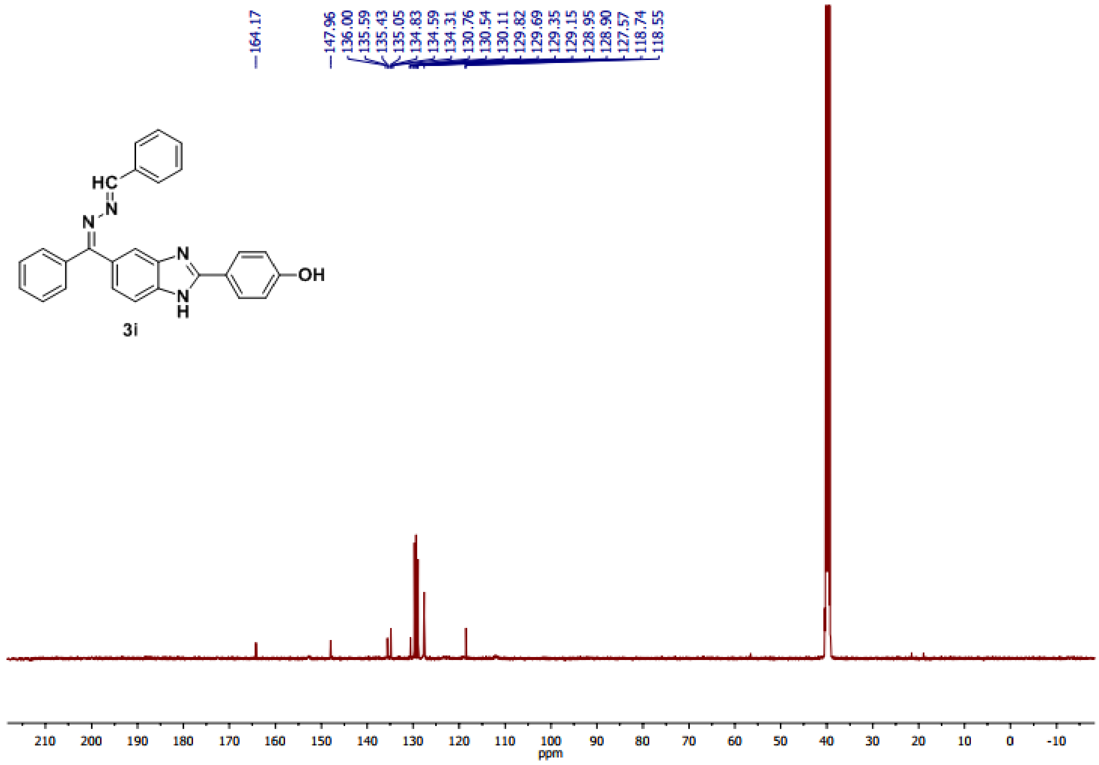

Figure S18: ^{13}C-NMR spectrum of compound **3i**

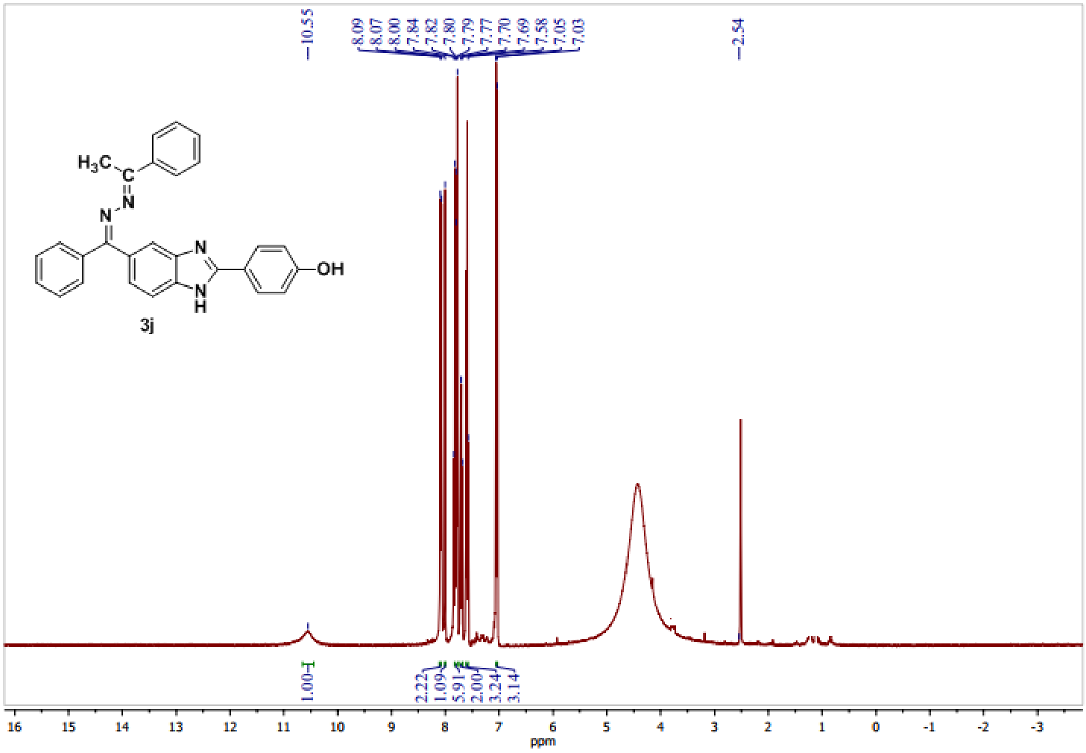

Figure S19: ^{1}H-NMR spectrum of compound **3j**

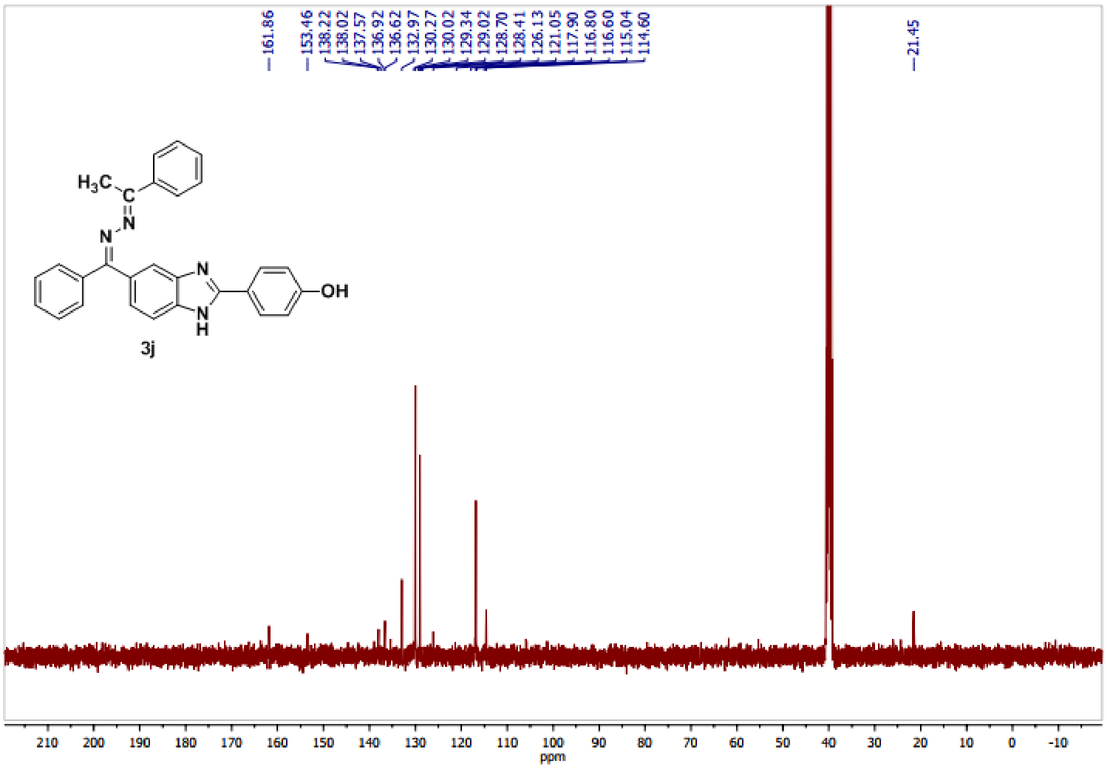

Figure S20: ^{13}C-NMR spectrum of compound **3j**

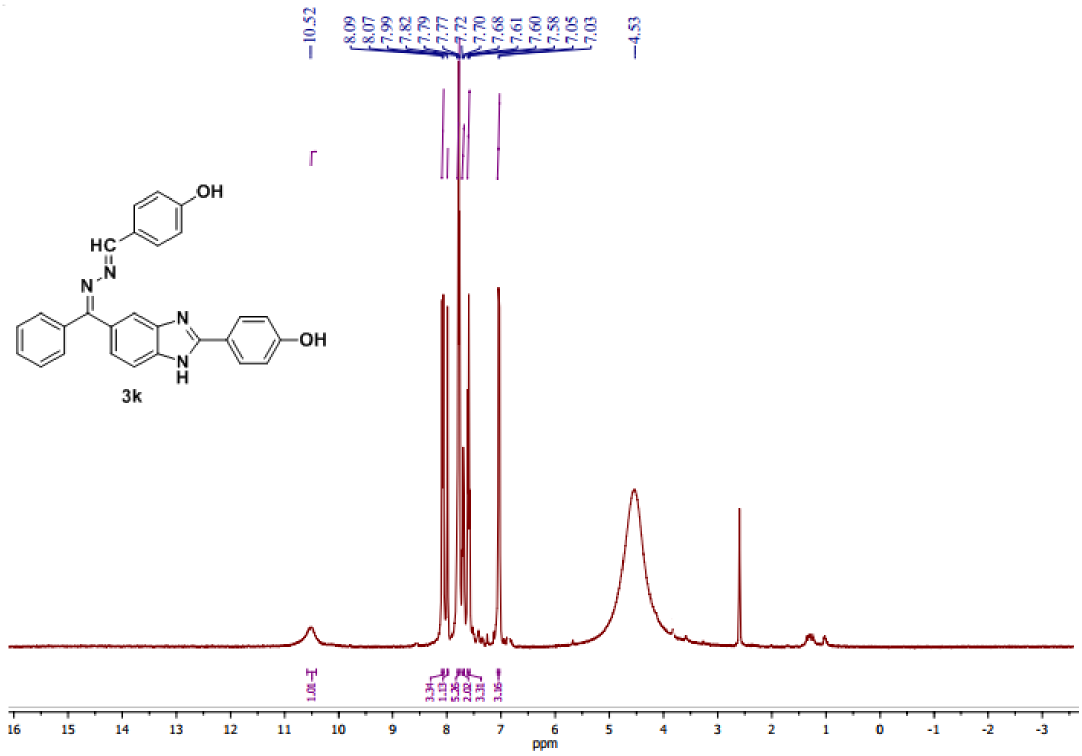

Figure S21: ^{1}H-NMR spectrum of compound **3k**

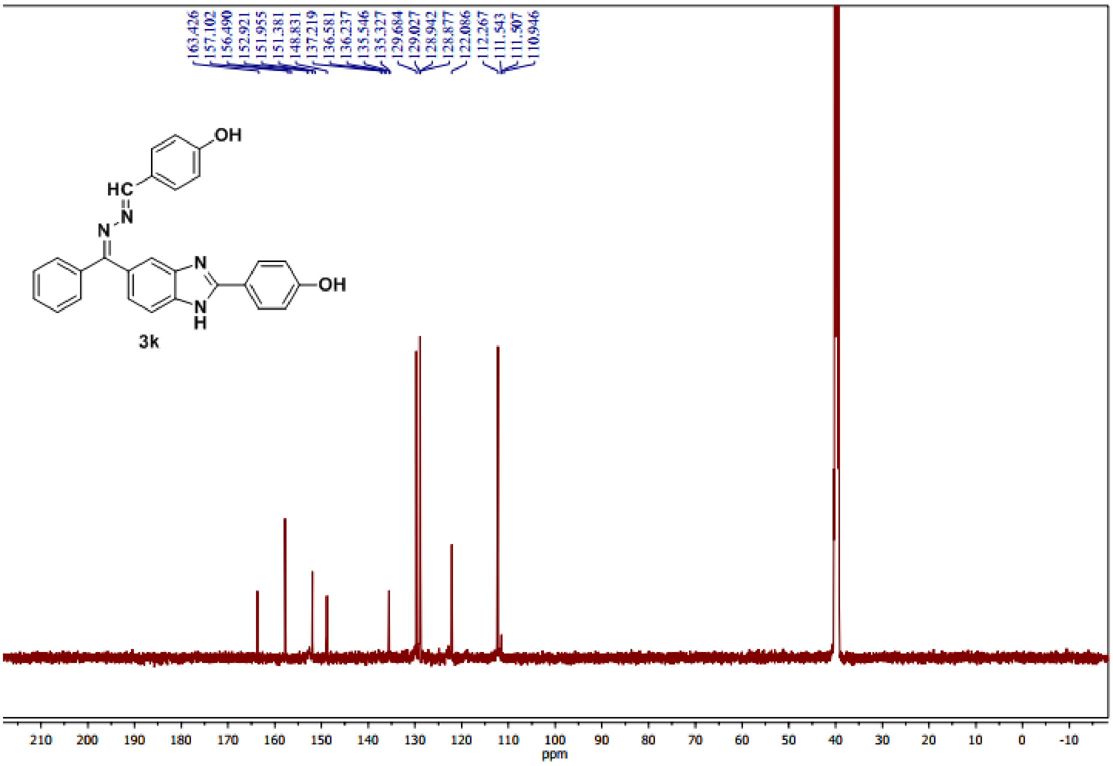

Figure S22: ^{13}C-NMR spectrum of compound **3k**

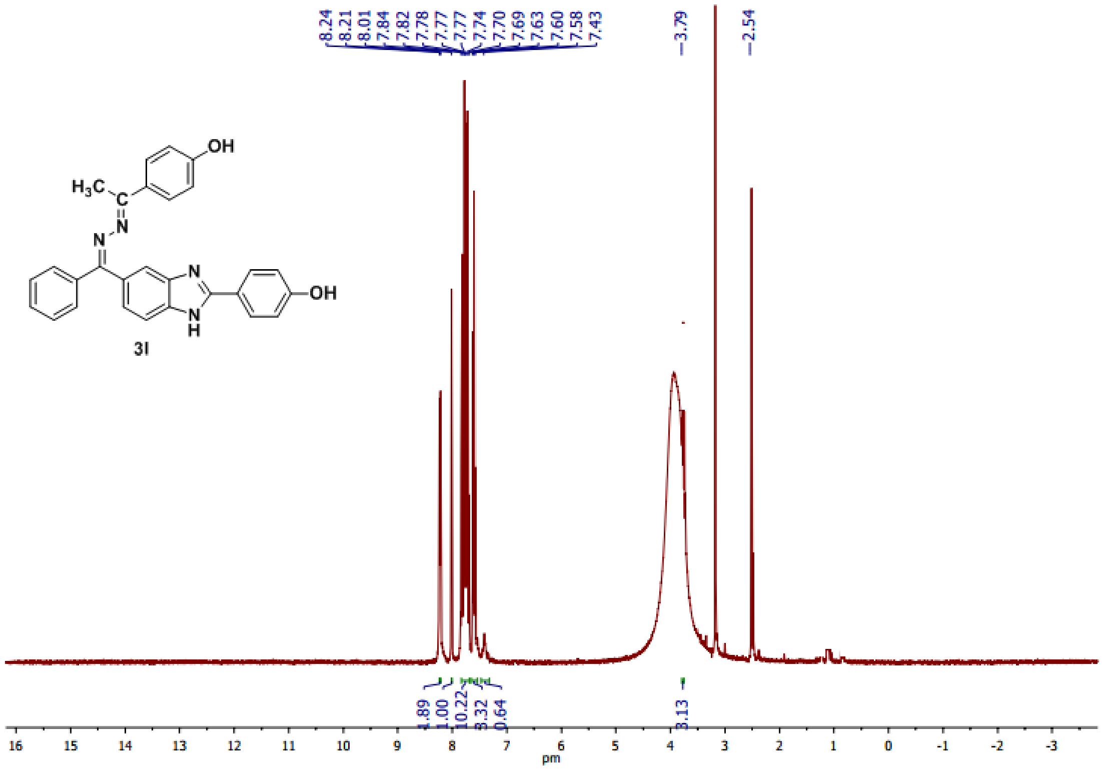

Figure S23: ^{1}H-NMR spectrum of compound **3l**

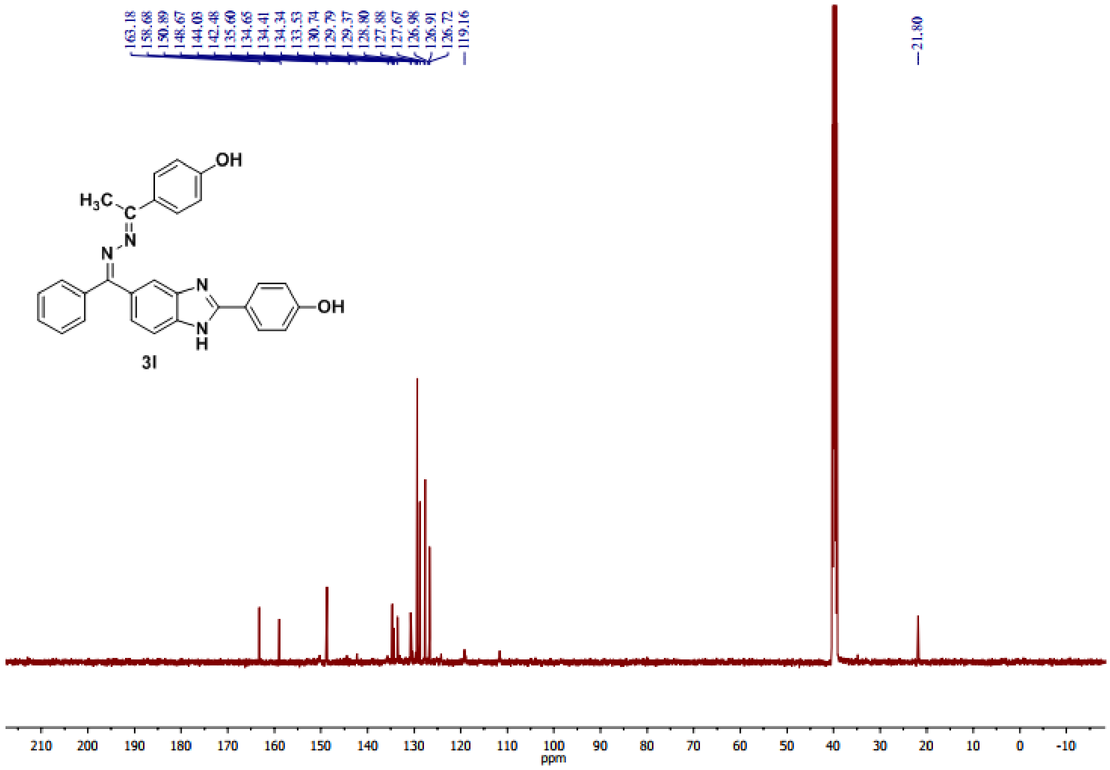

Figure S24: ^{13}C-NMR spectrum of compound **3l**

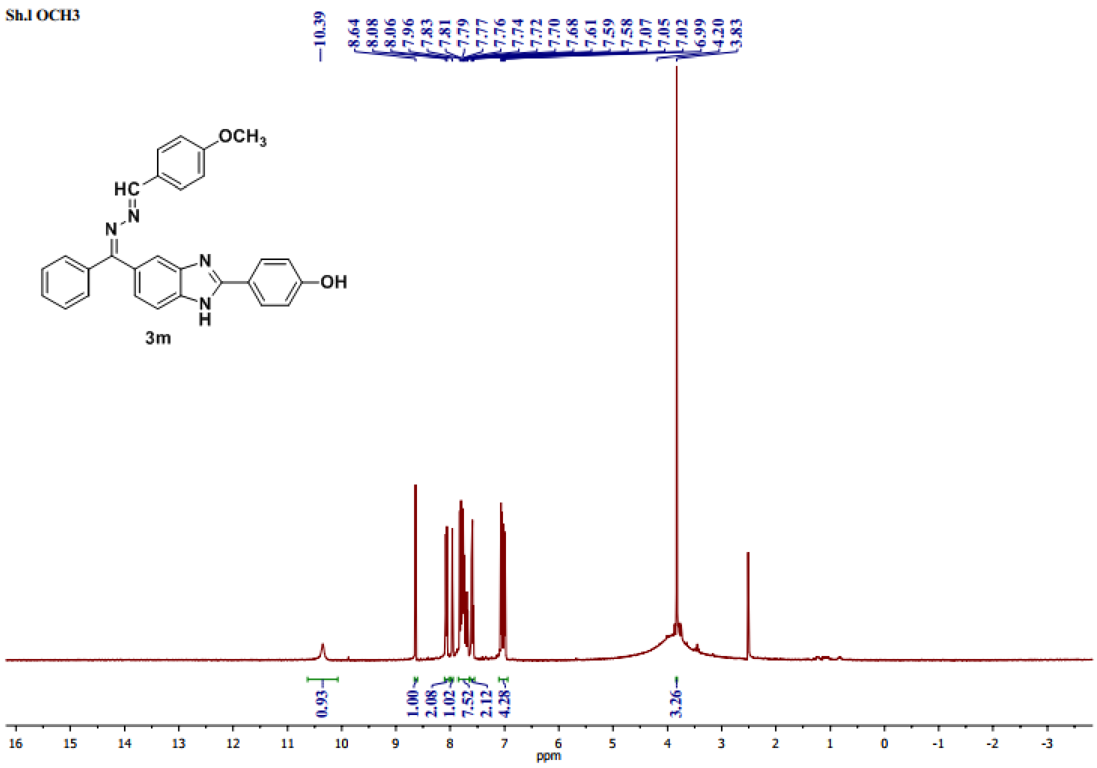

Figure S25: ^{1}H-NMR spectrum of compound **3m**

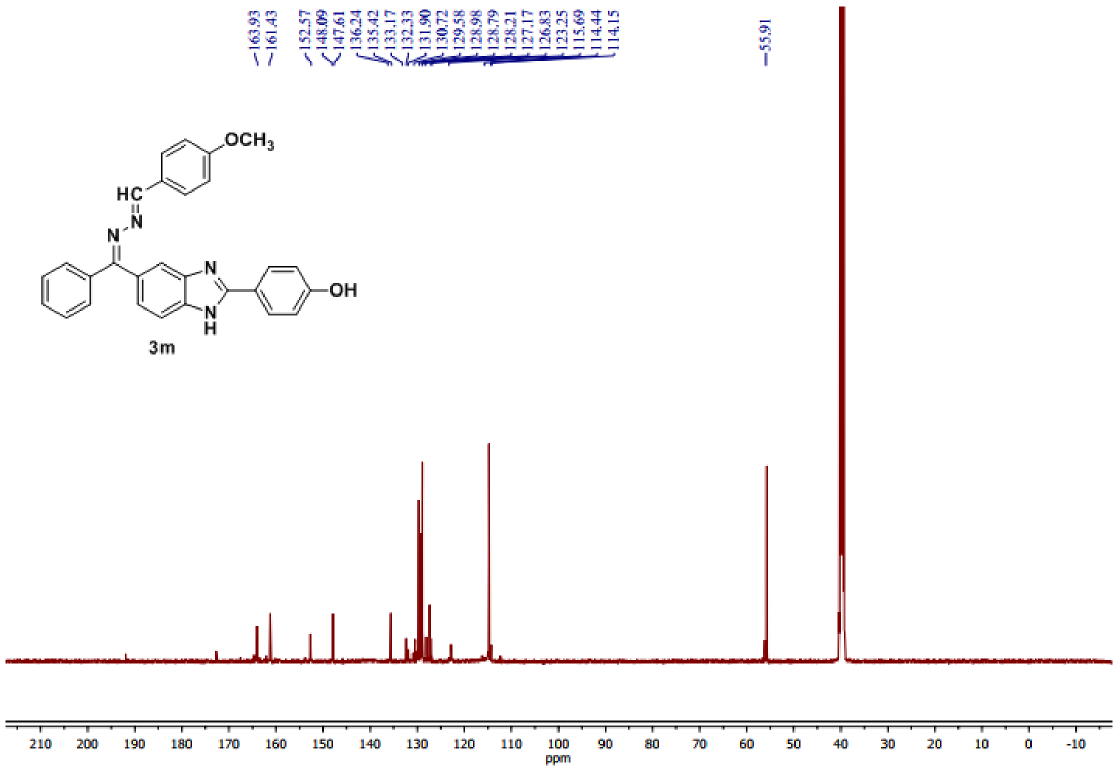

Figure S26: ^{13}C-NMR spectrum of compound **3m**

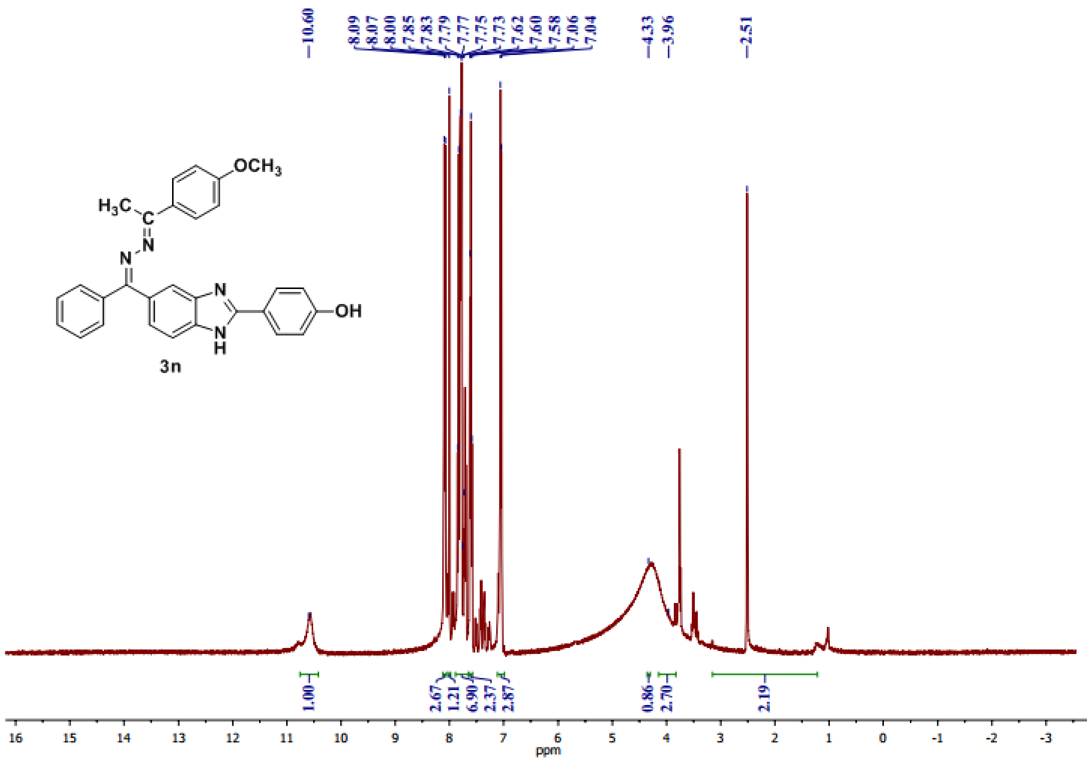

Figure S27: ^{1}H-NMR spectrum of compound **3n**

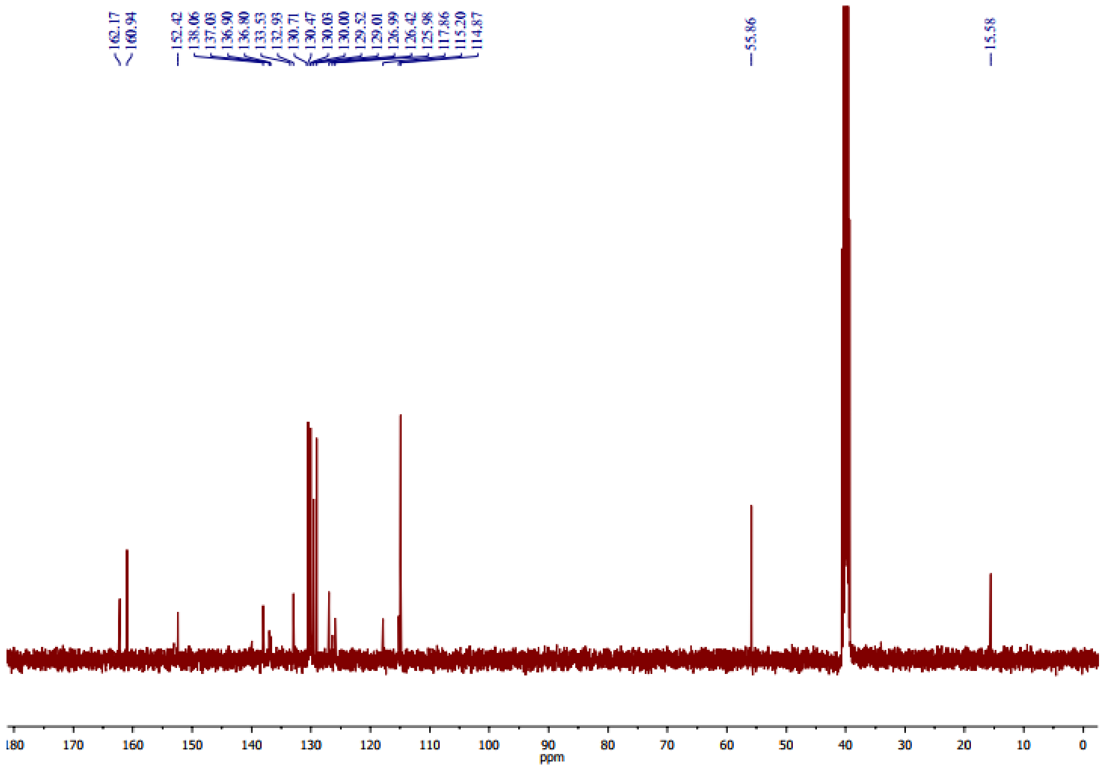

Figure S28: ^{13}C-NMR spectrum of compound **3n**

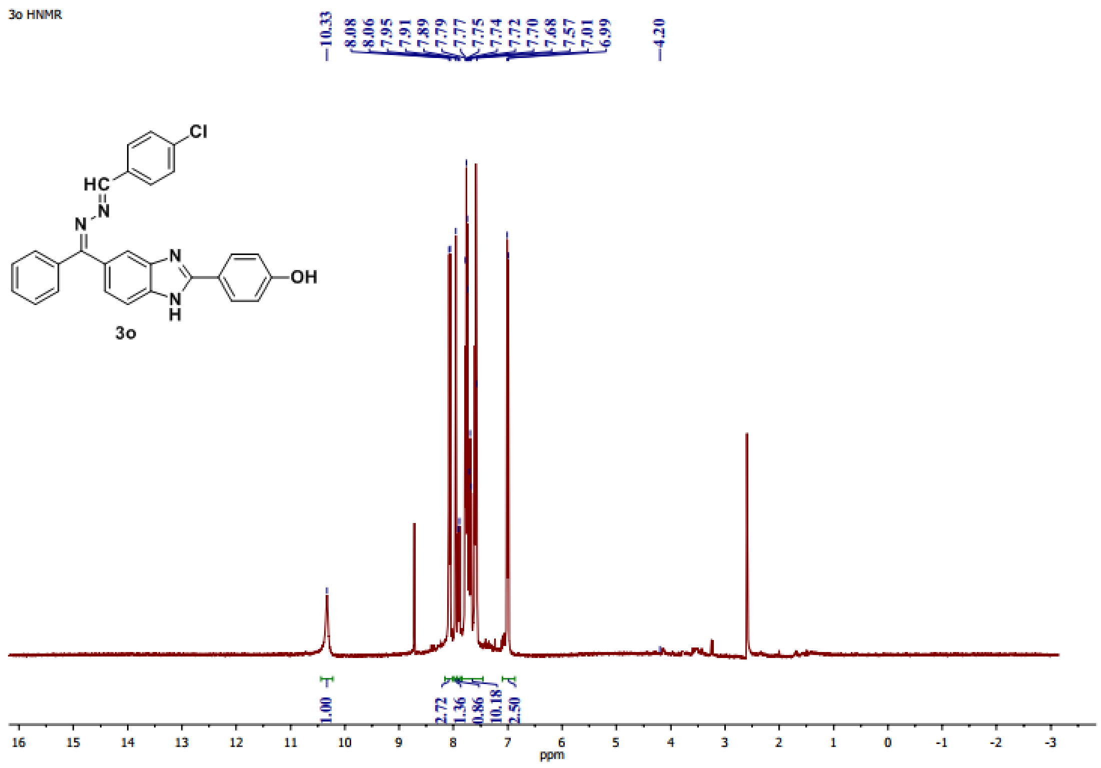

Figure S29: ^{1}H-NMR spectrum of compound **3o**

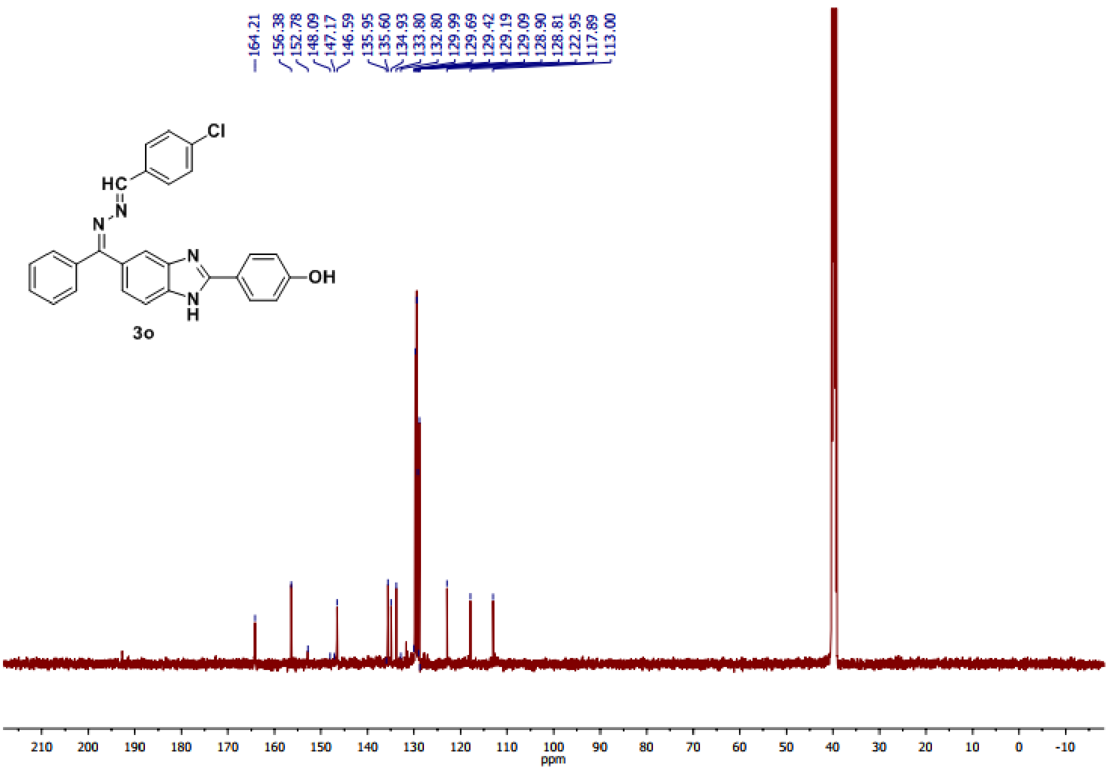

Figure S30: ^{13}C-NMR spectrum of compound **3o**

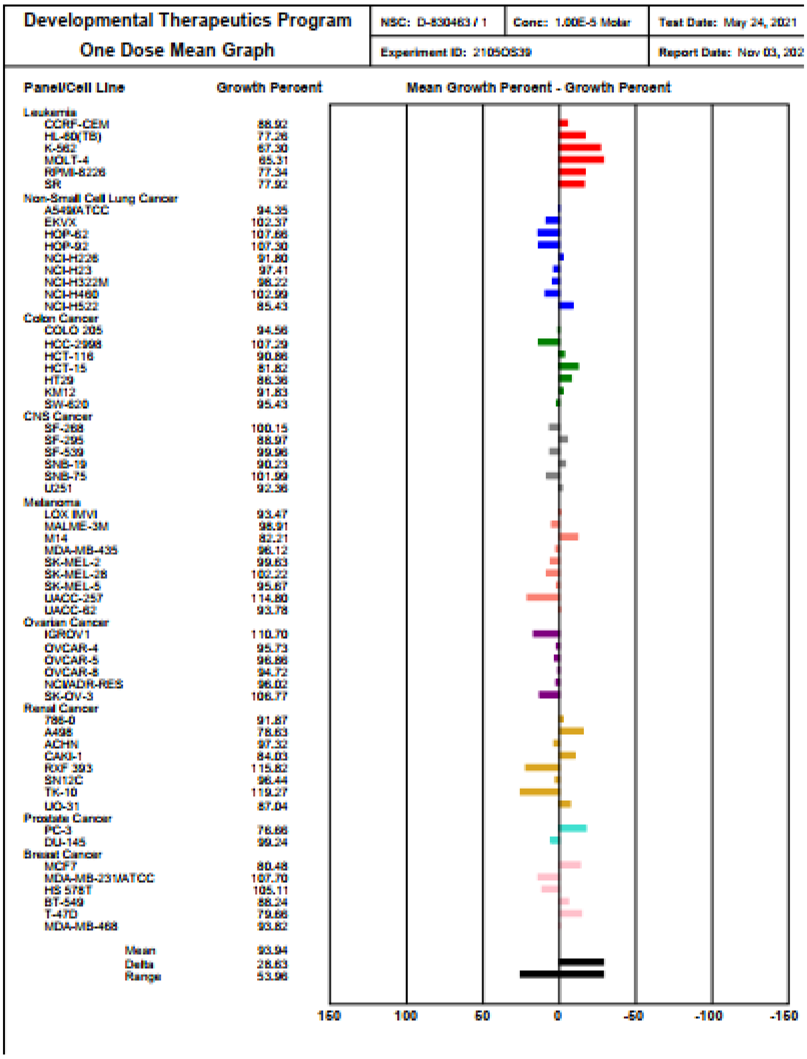

Figure S31: Results of primary *in vitro* one-dose anticancer assay of compound **3a.**

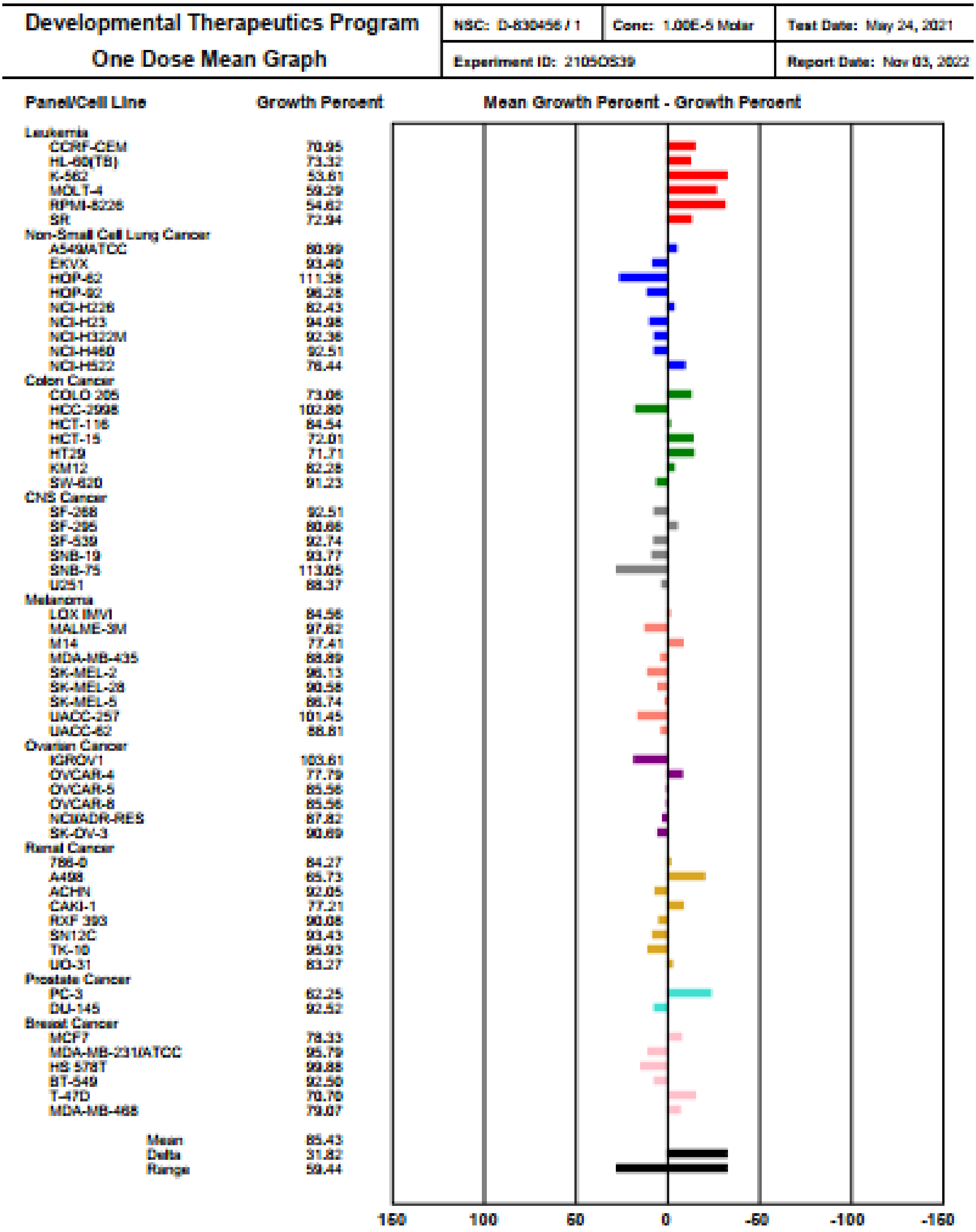

Figure S32: Results of primary *in vitro* one-dose anticancer assay of compound **3b.**

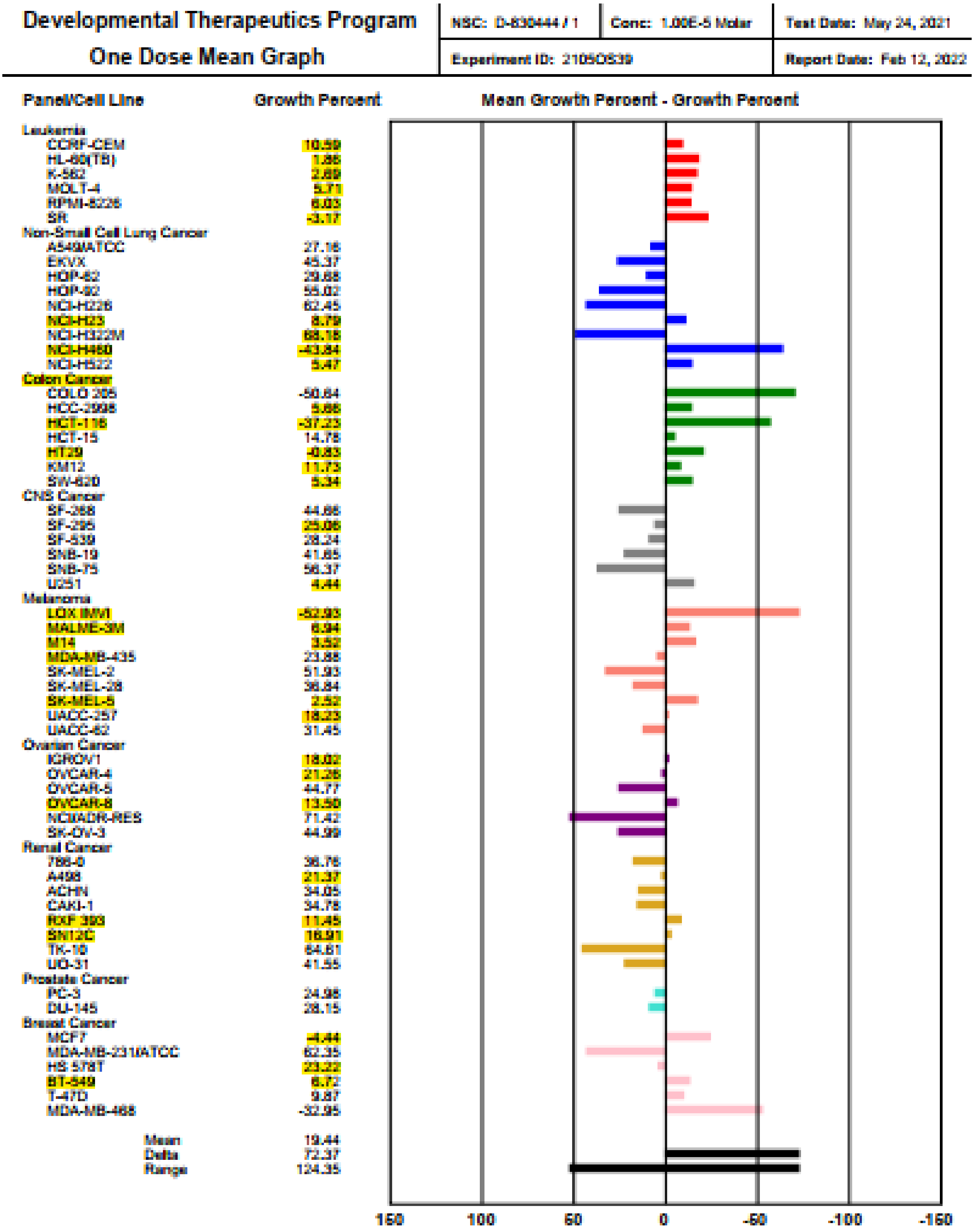

Figure S33: Results of primary *in vitro* one-dose anticancer assay of compound **3c.**

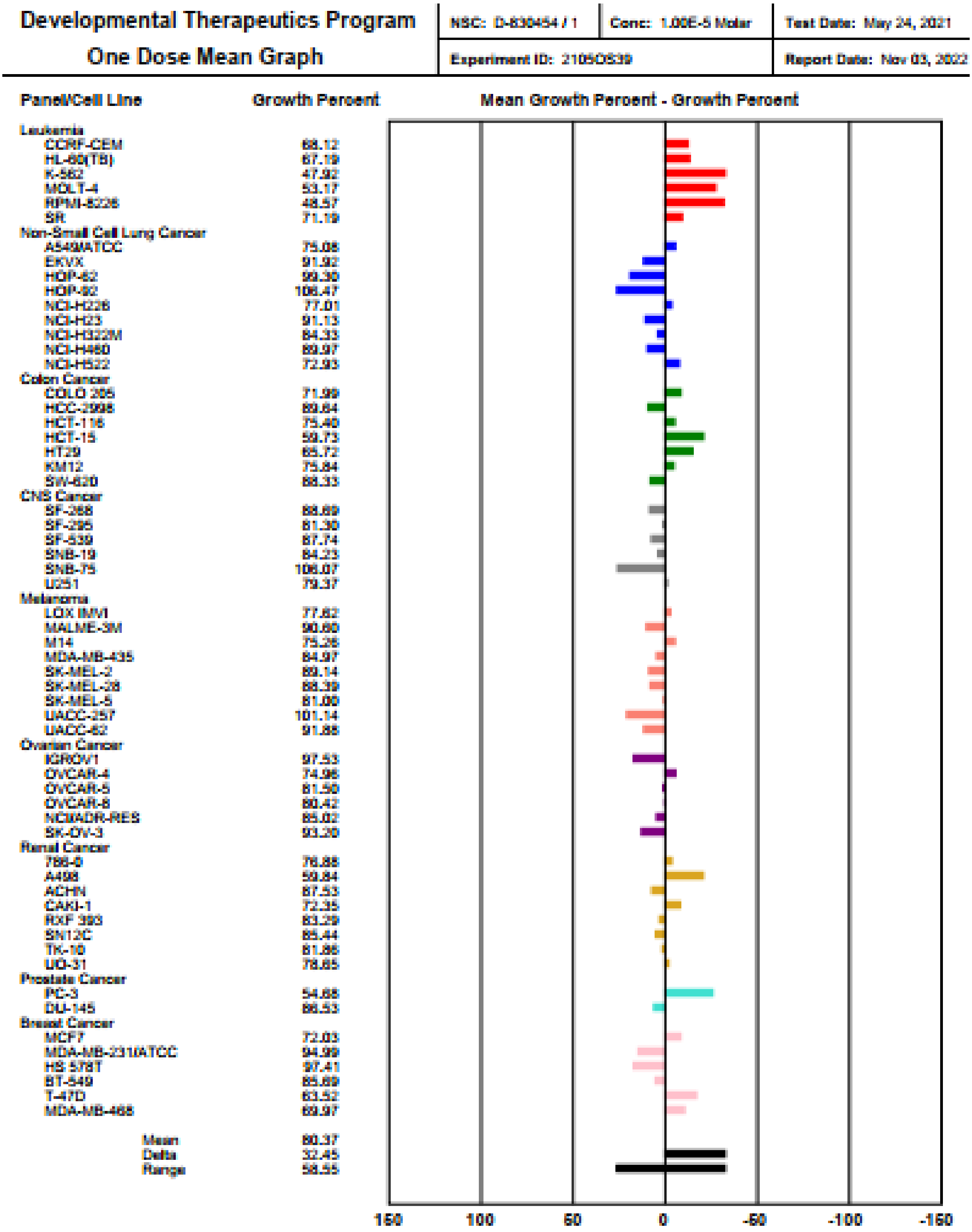

Figure S34: Results of primary *in vitro* one-dose anticancer assay of compound **3d.**

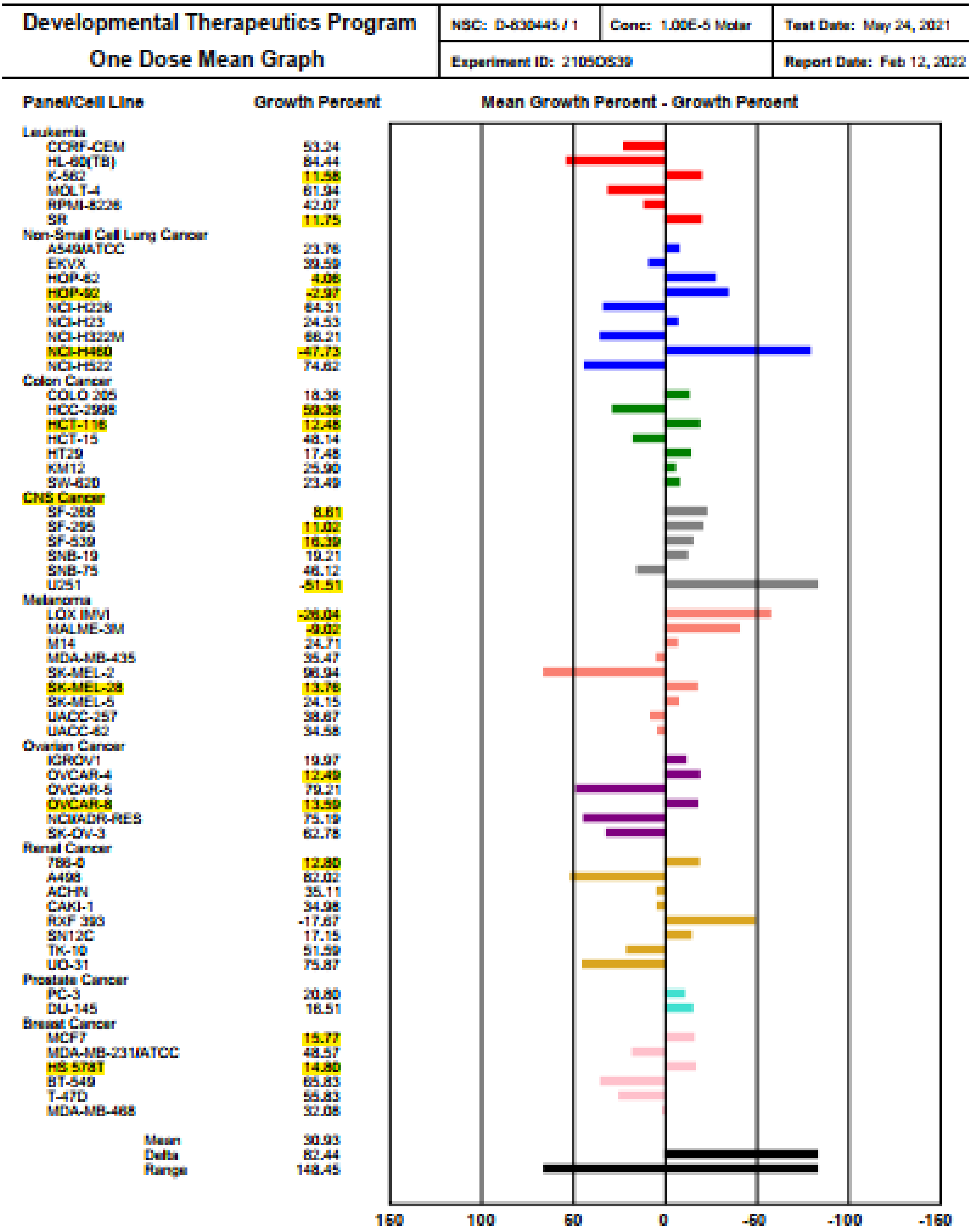

Figure S35: Results of primary *in vitro* one-dose anticancer assay of compound **3e.**

Developmental Therapeutics Program

One Dose Mean Graph

NSC: D-830455 / 1	Conc: 1.00E-5 Molar	Test Date: May 24, 2021
Experiment ID: 2105OS39		Report Date: Nov 03, 2022

Panel/Cell Line	Growth Percent
**Leukemia**	
CCRF-CEM	61.31
HL-60(TB)	65.43
K-562	36.82
MOLT-4	48.68
RPMI-8226	44.90
SR	63.05
**Non-Small Cell Lung Cancer**	
A549/ATCC	75.73
EKVX	90.47
HOP-62	101.17
HOP-92	94.98
NCI-H226	82.96
NCI-H23	91.75
NCI-H322M	94.50
NCI-H460	85.89
NCI-H522	71.24
**Colon Cancer**	
COLO 205	62.07
HCC-2998	83.10
HCT-116	66.71
HCT-15	45.08
HT29	57.81
KM12	75.48
SW-620	88.70
**CNS Cancer**	
SF-268	86.45
SF-295	79.95
SF-539	91.73
SNB-19	83.51
SNB-75	108.62
U251	75.38
**Melanoma**	
LOX IMVI	85.73
MALME-3M	95.04
M14	67.17
MDA-MB-435	84.83
SK-MEL-2	85.26
SK-MEL-28	90.99
SK-MEL-5	81.33
UACC-257	94.46
UACC-62	90.52
**Ovarian Cancer**	
IGROV1	100.47
OVCAR-4	73.88
OVCAR-5	82.11
OVCAR-8	76.88
NCI/ADR-RES	83.99
SK-OV-3	88.49
**Renal Cancer**	
786-0	68.37
A498	62.96
ACHN	85.67
CAKI-1	70.08
RXF 393	77.37
SN12C	87.21
TK-10	85.71
UO-31	75.44
**Prostate Cancer**	
PC-3	50.65
DU-145	81.64
**Breast Cancer**	
MCF7	70.76
MDA-MB-231/ATCC	101.91
HS 578T	92.01
BT-549	79.98
T-47D	56.17
MDA-MB-468	72.41
Mean	78.15
Delta	41.33
Range	69.80

Mean Growth Percent - Growth Percent

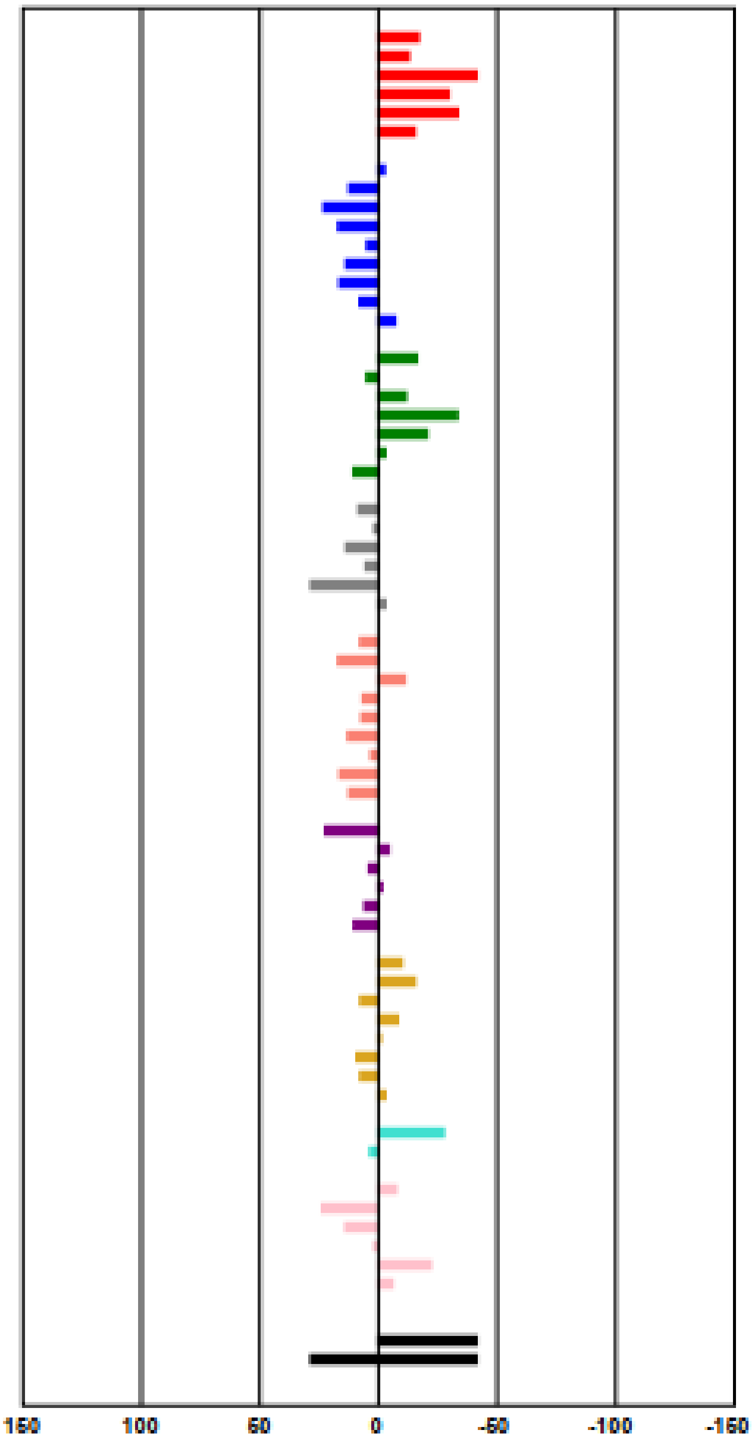

Figure S36: Results of primary *in vitro* one-dose anticancer assay of compound **3f.**

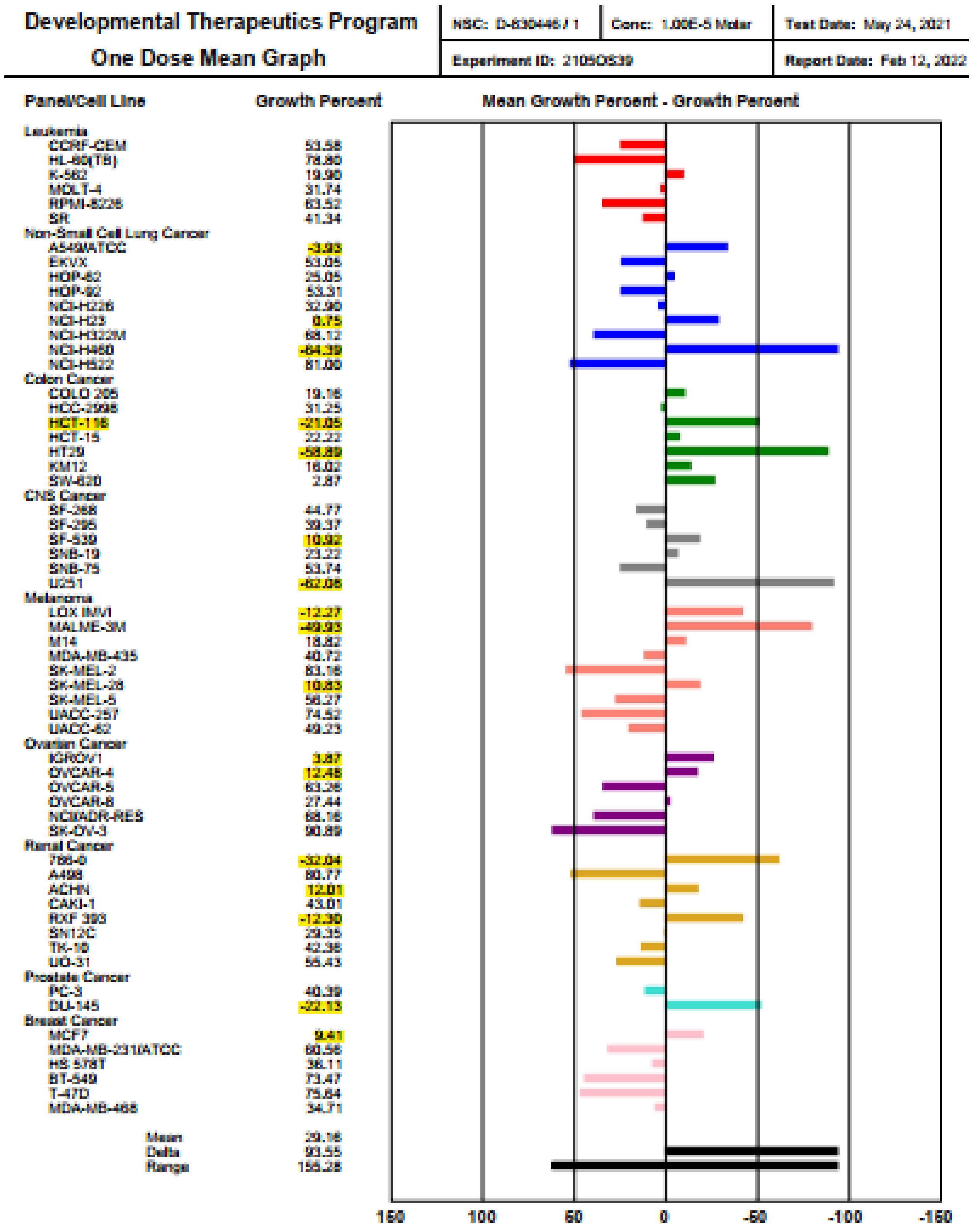

Figure S37: Results of primary *in vitro* one-dose anticancer assay of compound **3g.**

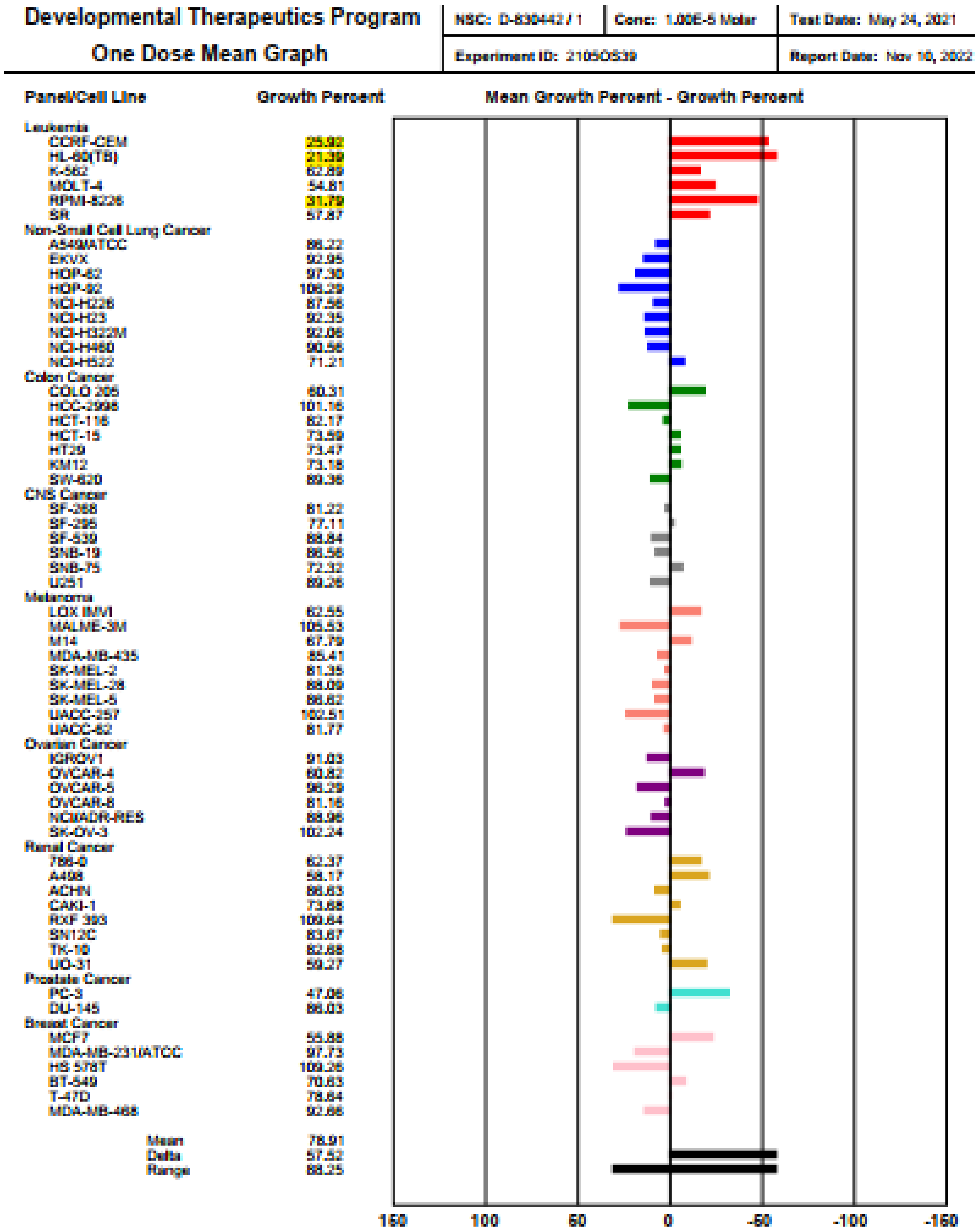

Figure S38: Results of primary *in vitro* one-dose anticancer assay of compound **3h.**

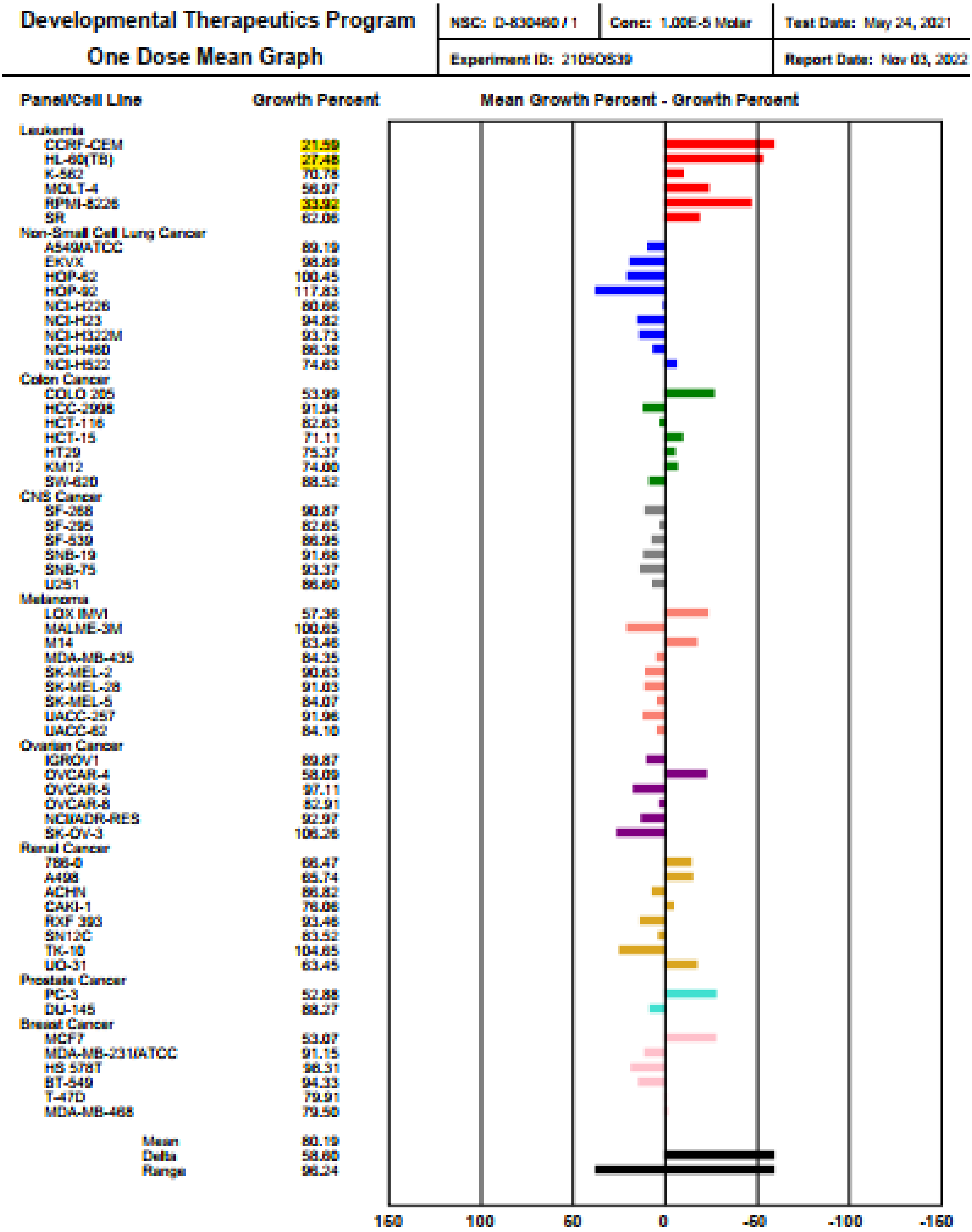

Figure S39: Results of primary *in vitro* one-dose anticancer assay of compound **3i.**

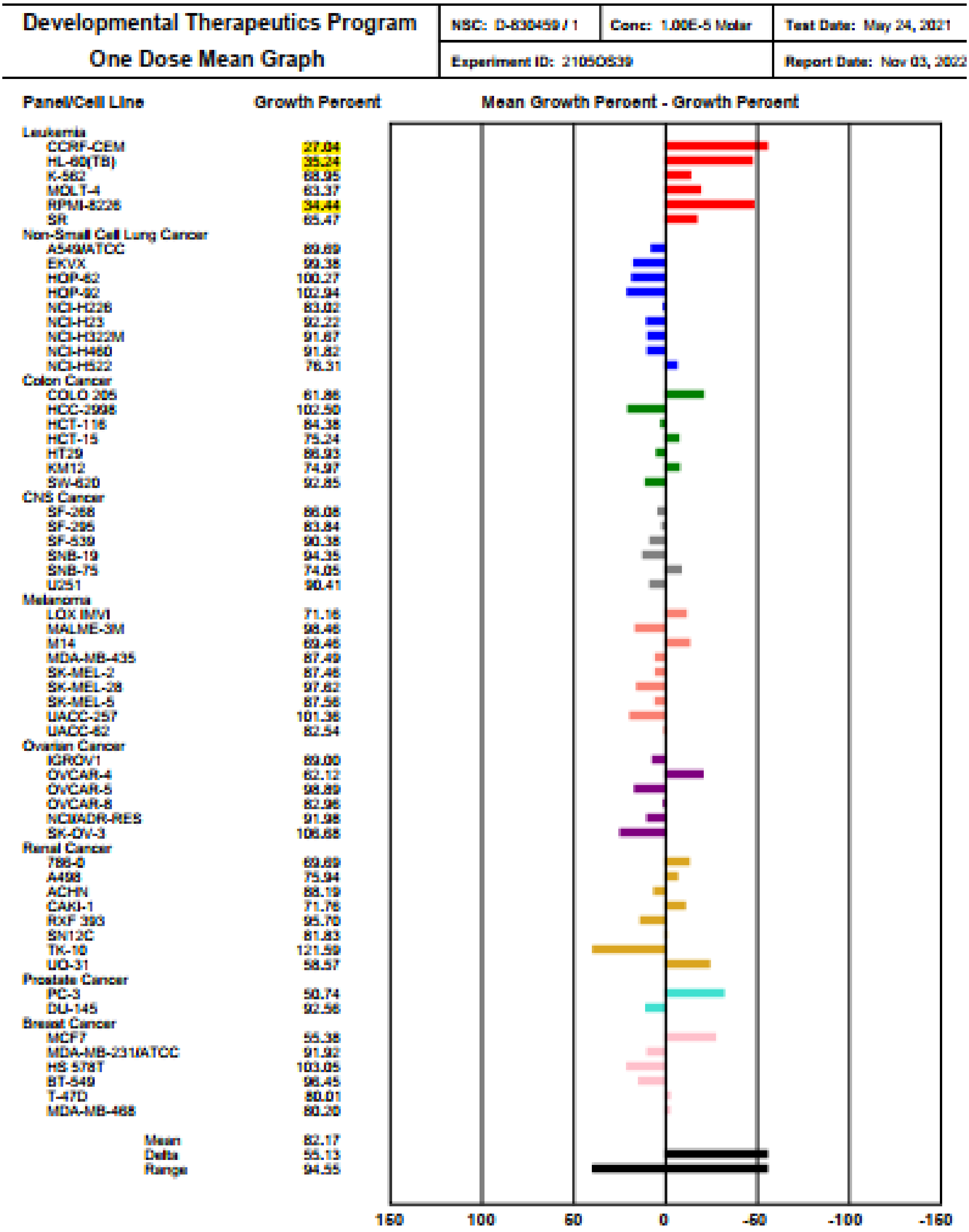

Developmental Therapeutics Program
One Dose Mean Graph

NSC: D-830459 / 1 | Conc: 1.00E-5 Molar | Test Date: May 24, 2021
Experiment ID: 2105OS39 | Report Date: Nov 03, 2022

Panel/Cell Line	Growth Percent
**Leukemia**	
CCRF-CEM	27.04
HL-60(TB)	35.24
K-562	68.95
MOLT-4	63.37
RPMI-8226	34.44
SR	65.47
**Non-Small Cell Lung Cancer**	
A549/ATCC	89.69
EKVX	99.38
HOP-62	100.27
HOP-92	102.94
NCI-H226	83.02
NCI-H23	92.22
NCI-H322M	91.67
NCI-H460	91.82
NCI-H522	76.31
**Colon Cancer**	
COLO 205	61.86
HCC-2998	102.50
HCT-116	84.38
HCT-15	75.24
HT29	86.93
KM12	74.97
SW-620	92.85
**CNS Cancer**	
SF-268	86.08
SF-295	83.84
SF-539	90.38
SNB-19	94.35
SNB-75	74.05
U251	90.41
**Melanoma**	
LOX IMVI	71.16
MALME-3M	98.46
M14	69.46
MDA-MB-435	87.49
SK-MEL-2	87.46
SK-MEL-28	97.62
SK-MEL-5	87.56
UACC-257	101.38
UACC-62	82.54
**Ovarian Cancer**	
IGROV1	89.00
OVCAR-4	62.12
OVCAR-5	98.89
OVCAR-8	82.96
NCI/ADR-RES	91.98
SK-OV-3	106.68
**Renal Cancer**	
786-0	69.69
A498	75.94
ACHN	88.19
CAKI-1	71.76
RXF 393	95.70
SN12C	81.83
TK-10	121.59
UO-31	58.57
**Prostate Cancer**	
PC-3	50.74
DU-145	92.56
**Breast Cancer**	
MCF7	55.38
MDA-MB-231/ATCC	91.92
HS 578T	103.05
BT-549	96.45
T-47D	80.01
MDA-MB-468	80.20
Mean	82.17
Delta	55.13
Range	94.55

Figure S40: Results of primary *in vitro* one-dose anticancer assay of compound **3j.**

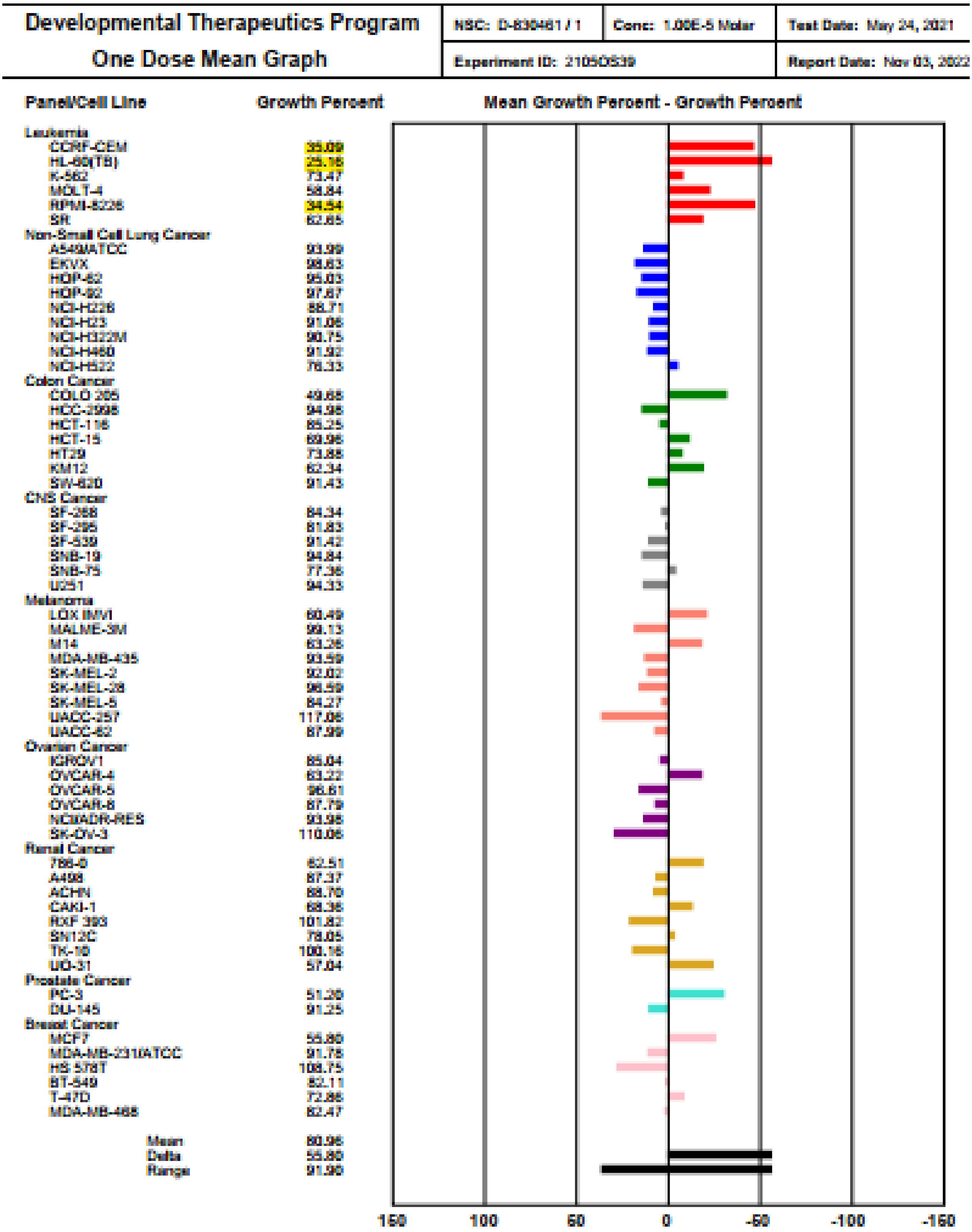

Figure S41: Results of primary *in vitro* one-dose anticancer assay of compound **3k.**

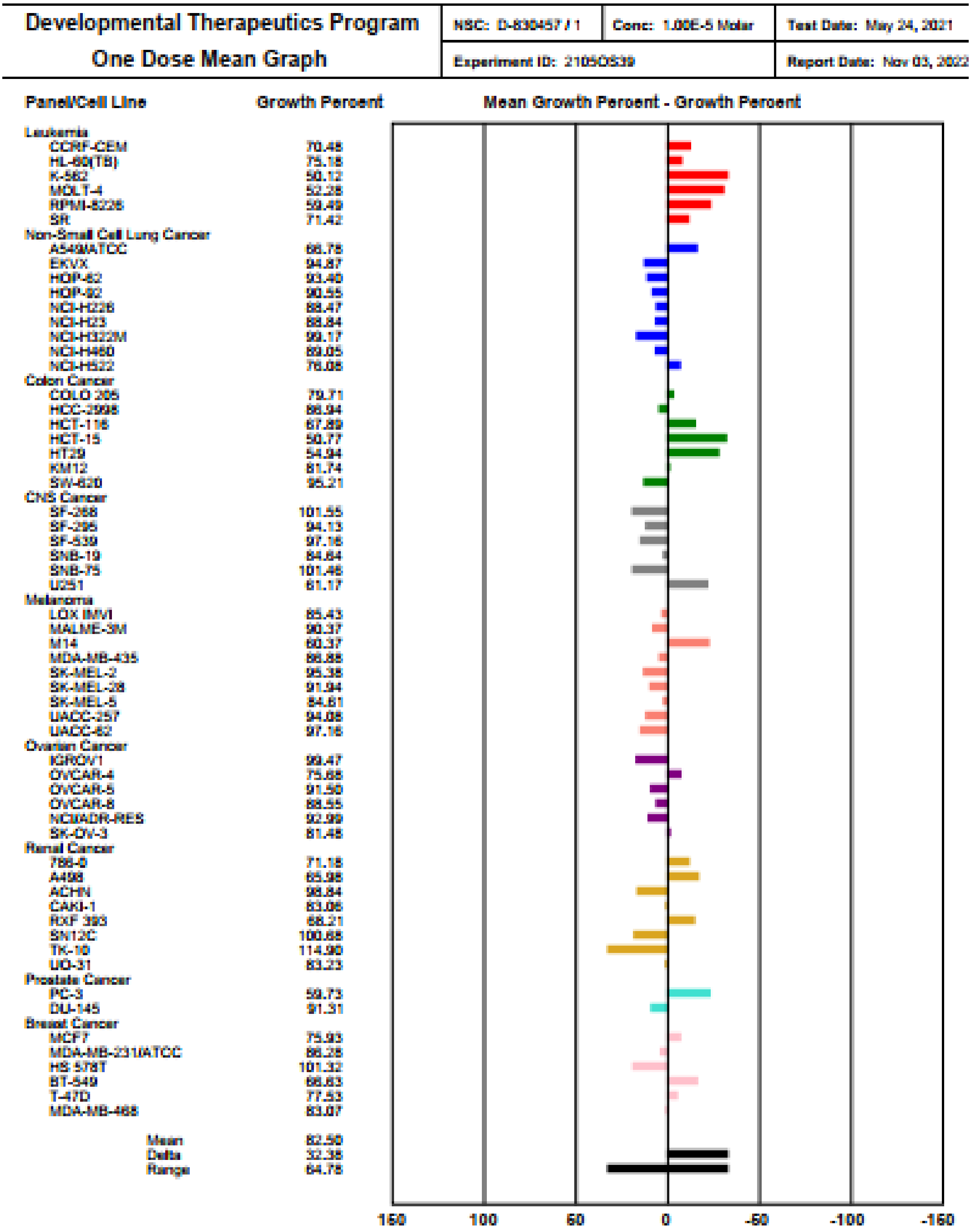

Figure S42: Results of primary *in vitro* one-dose anticancer assay of compound **3l.**

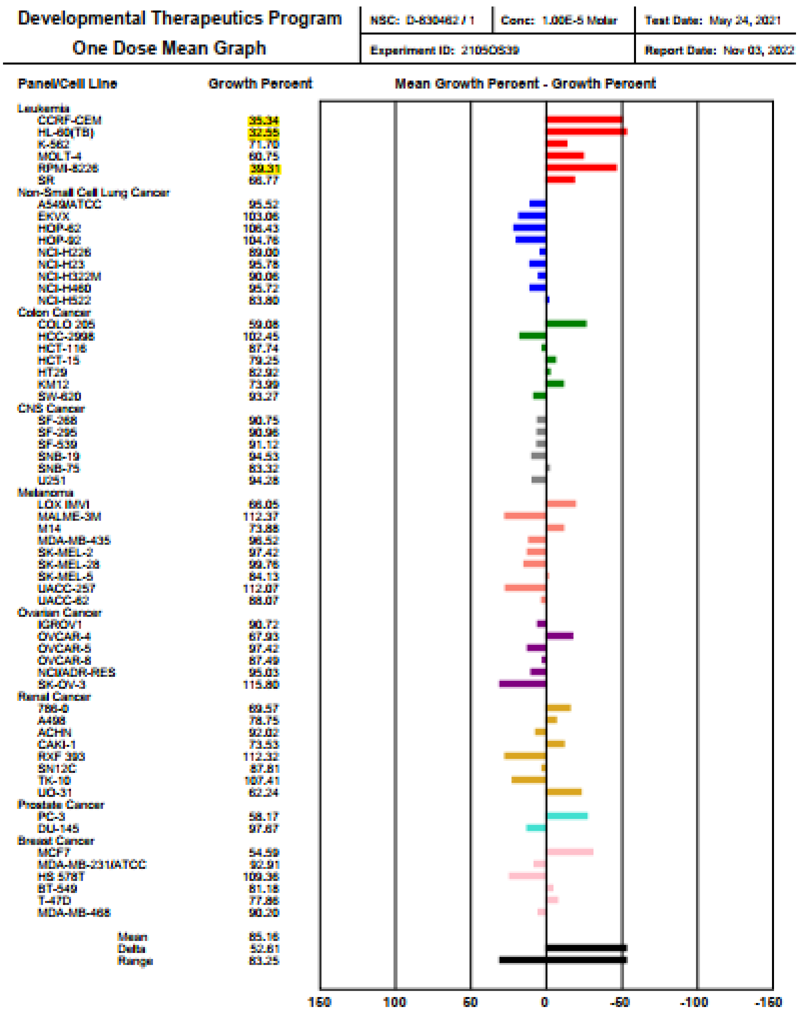

Figure S43: Results of primary *in vitro* one-dose anticancer assay of compound **3m.**

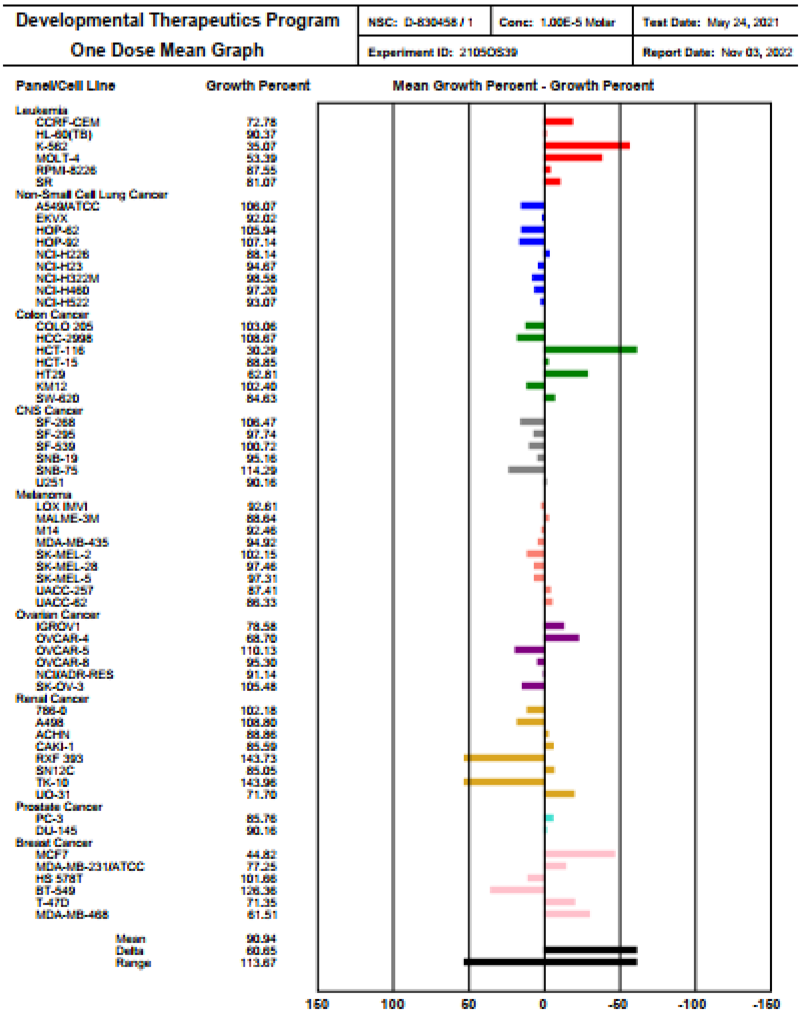

Figure S44: Results of primary *in vitro* one-dose anticancer assay of compound **3n.**

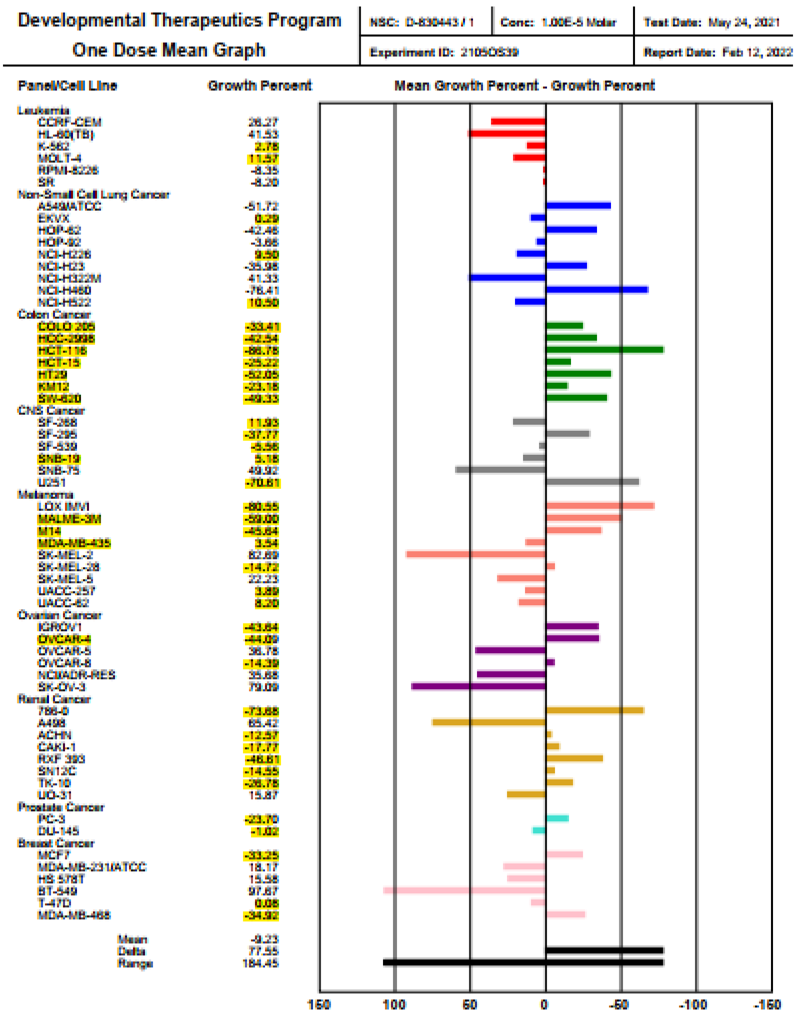

Figure S45: Results of primary *in vitro* one-dose anticancer assay of compound **3o.**

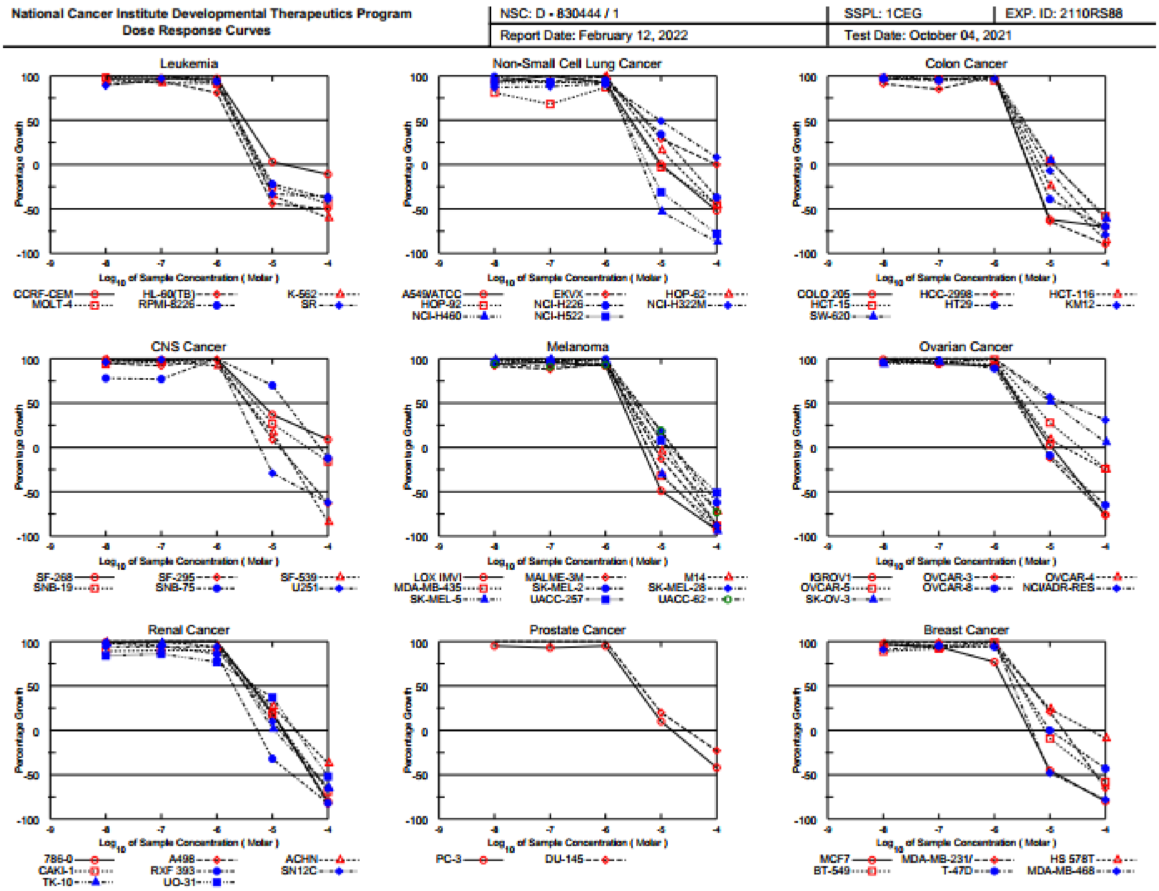

Figure S46: Dose response curve of the *in vitro* NCI five dose anticancer assay of compound **3c**.

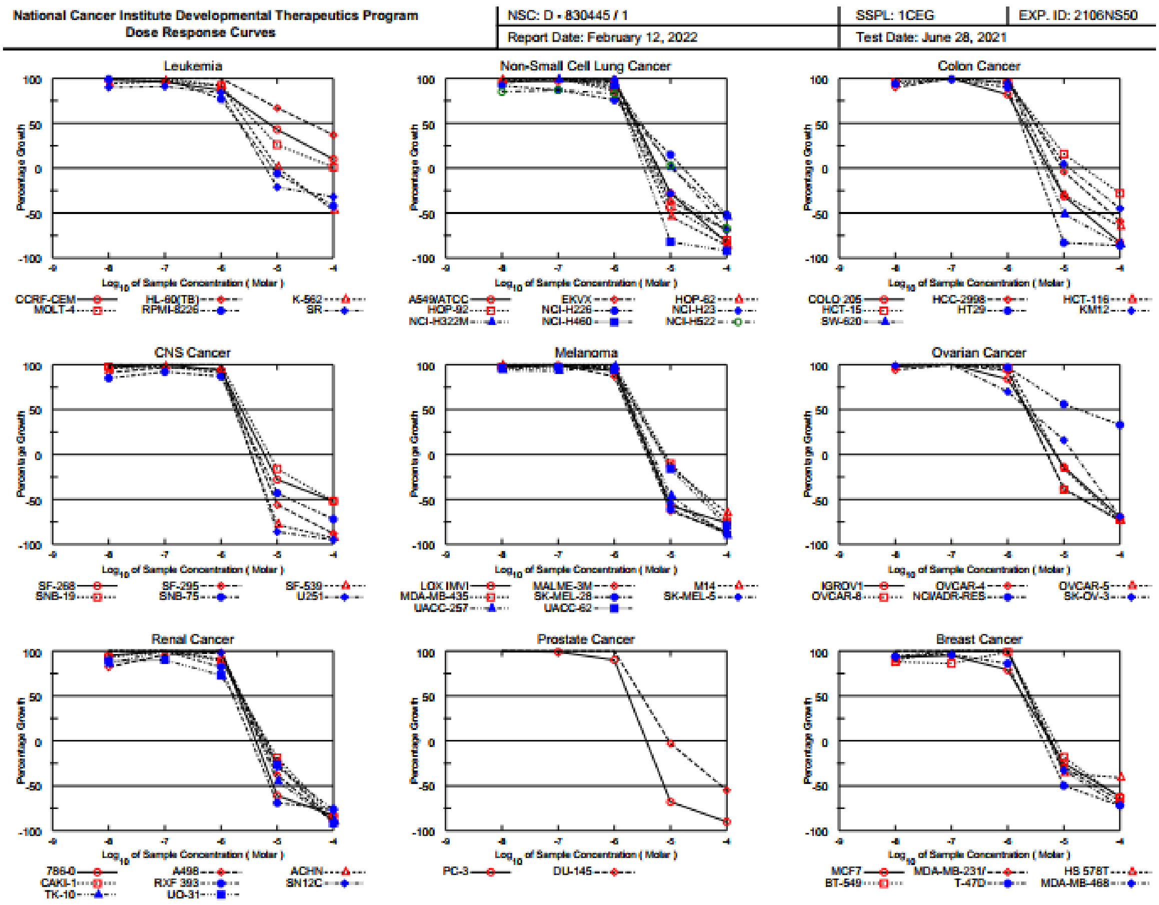

Figure S47: Dose response curve of the *in vitro* NCI five dose anticancer assay of compound **3e**.

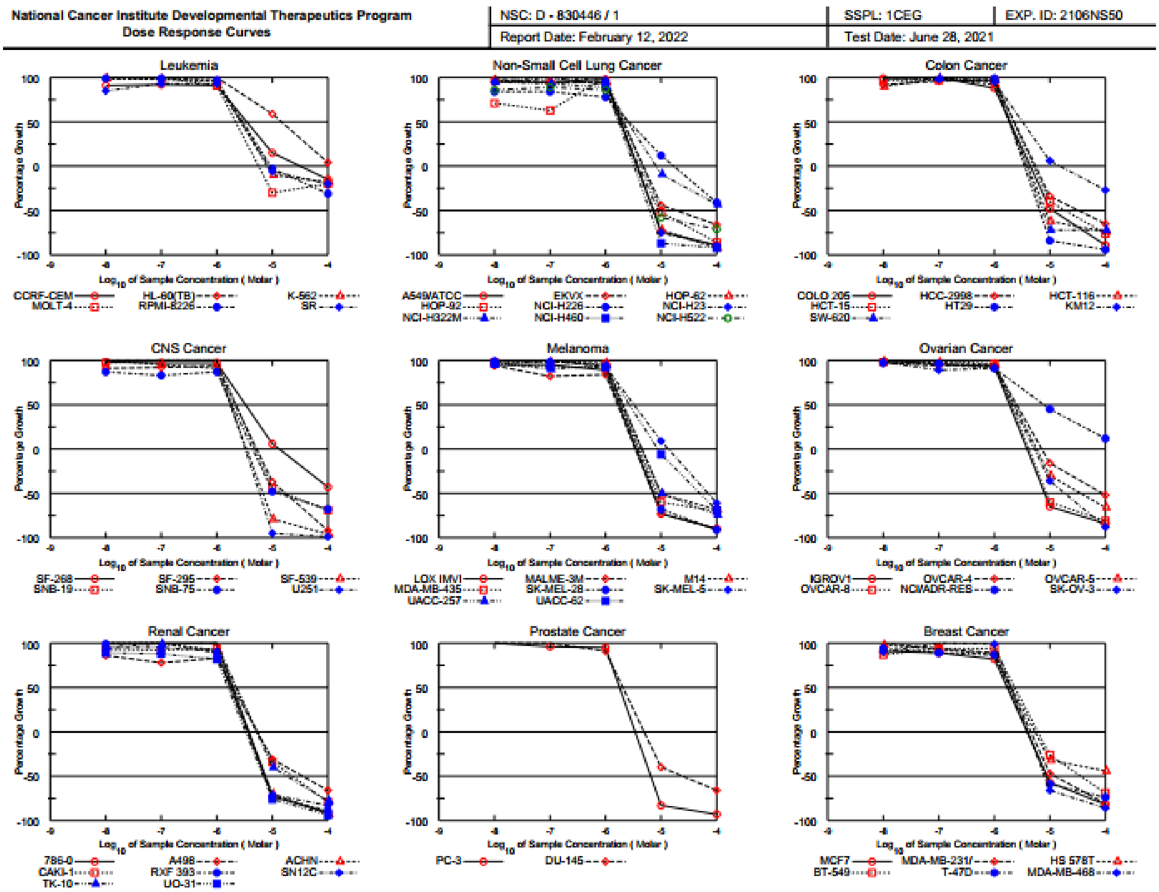

Figure S48: Dose response curve of the *in vitro* NCI five dose anticancer assay of compound **3g**.

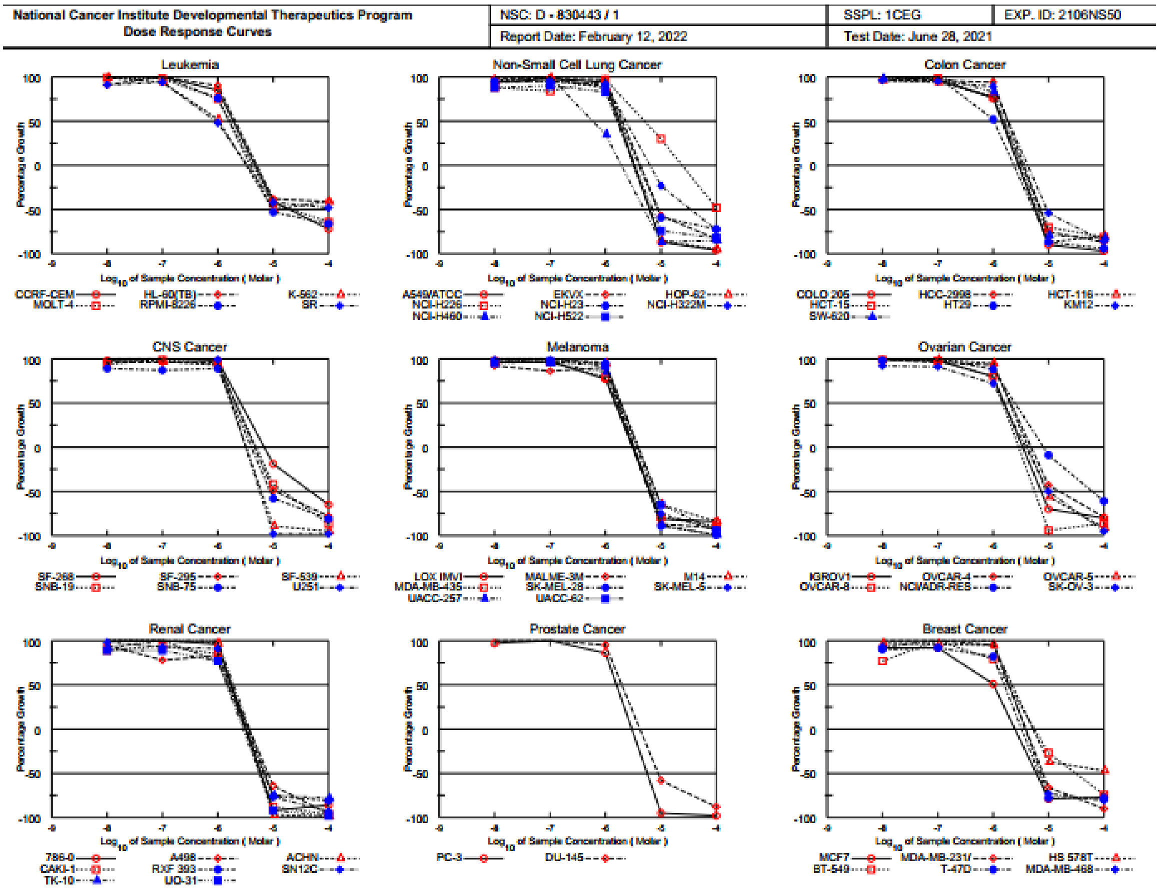

Figure S49: Dose response curve of the *in vitro* NCI five dose anticancer assay of compound **3o**.

Appendix A

4.2. Biological studies

4.1.1. Cytotoxic activity evaluation against lung cancer cell lines

To determine IC_{50}, benzimidazole molecules **3c, 3e, 3g** and **3o** were further analyzed using the standard MTT colorimetric assay against lung cancer A549 and NCI-H460 cell lines. Cells at density of 1 x 10^4 were seeded in a 96-well plate at 37 ^oC for 24 h under 5% CO_2. After incubation, the cells were treated with different concentrations of the test compounds **3c, 3e, 3g** and **3o** and incubated for 24 h, then 20 μl of MTT solution at 5 mg/mL was applied and incubated for 4 h at 37 °C. Dimethyl sulphoxide (DMSO) in volume of 100 µl was added to each well to dissolve the purple formazan that had formed. The color intensity of the formazan product, which represents the growth condition of the cells, is quantified by using an ELISA plate reader (EXL 800, USA) at 570 nm absorbance. The experimental conditions were carried out with at least three replicates, and the experiments were repeated at least three times.

4.2.2. VEGFR-2 inhibition assay

Benzimidazole compounds **3e, 3g** and Sorafenib were evaluated for their VEGFR-2 inhibitory activity according to manufacturer's instructions using # VEGFR-2 (KDR) Kinase Assay Kit Catalog # 40325 (BPS Bioscience).

4.2.3. Cell cycle analysis of compounds 3e and 3g

Cell cycle analysis in NCI-H460 cells was investigated using fluorescent Annexin V-FITC/ PI detection kit (*BioVision* EZCell^{TM} Cell Cycle Analysis Kit Catalog #K920) by flow cytometry assay. NCI-H460 cells at a density of 2 × 10^5 per well were harvested and washed twice in PBS. After that, the cells were incubated at 37 °C and 5% CO_2. The medium was incubated with the tested compounds **3e** and **3g** at their IC_{50} (μM) for 24 h, washed twice in PBS, fixed with 70% ethanol, rinsed again with PBS. Afterward,

medium was stained with DNA fluorochrome PI for 15 min at 37 °C. The samples were immediately analyzed using Facs Calibur flow cytometer (Becton and Dickinson, Heidelberg, Germany).

4.2.4. Apoptosis assay for compounds 3e and 3g

Apoptosis in NCI-H460 cells was investigated using fluorescent Annexin V-FITC/ PI detection kit (*BioVision* Annexin V-FITC Apoptosis Detection Kit, Catalog #: K101) by flow cytometry assay. NCI-H460 cells at a density of 2×10^5 per well were treated with compounds **3e** and **3g** at their IC_{50} (μM) for 24 h, then the cells were harvested and stained with Annexin V-FITC/ PI dye for 15 min in the dark at 37 °C. The samples were immediately analyzed using *FACS Calibur* flow cytometer (Becton and Dickinson, Heidelberg, Germany).

4.2.5. *C*aspase 9 assay for compounds 3e and 3g

To determine the effect of the synthesized benzimidazole hybrids **3e** and **3g** on apoptosis, the active *c*aspase 9 level was measured using ELISA analysis according to manufacturer's instructions. Briefly, NCI-H460 cells at a density of 2×10^4 per well were treated with compounds **3e** and **3g** at their IC_{50} (μM) for 24 h, then the cells were lysed with cell extraction buffer. This lysate was diluted in standard diluent buffer over the range of the assay. The optical density of each well was determined within 30 min using a microplate plate reader set at 450 nm to determine the human active *c*aspase 9 level.

4.2.6. Molecular modeling study

Docking studies, calculations, and investigations was conducting using Molecular Operating Environment MOE version MOE (2021.01.01 Chemical Computing Group, Canada Software) to perform molecular modeling study. Structure of **3g** was built in database of MOE. The X-ray crystal structure of Sorafenib bound to the VEGFR-2

enzyme binding site (PDB ID: 4ASD) active site was acquired from protein data bank at research collaboration for Structural Bioinformatics (RSCB) protein database [PDB].
